# The shifting global landscape of low Back pain attributable to high body mass index: Burden, growth, and inequalities

**DOI:** 10.1016/j.pmedr.2025.103031

**Published:** 2025-03-12

**Authors:** Fan Wang, Yisen Yang, Jing Xu, Meiduo Zhao, Hongwei Ma, Qun Xu

**Affiliations:** a School of Population Medicine and Public Health, Chinese Academy of Medical Sciences & Peking Union Medical College, Beijing 100730, China; b Department of Epidemiology and Biostatistics, Institute of Basic Medical Sciences Chinese Academy of Medical Sciences, School of Basic Medicine Peking Union Medical College, Beijing 100005, China; c Center of Environmental and Health Sciences, Chinese Academy of Medical Sciences, Peking Union Medical College, Beijing 100005, China; d Department of Rehabilitation Medicine & Department of Pain Medicine, Peking University International Hospital & PKUCare Rehabilitation Hospital, Beijing 102206, China; e Center for Rare Diseases, State Key Laboratory of Complex, Severe, and Rare Diseases, Peking Union Medical College Hospital, Chinese Academy of Medical Sciences, Beijing 100730, China

**Keywords:** Low back pain, Body mass index, Global burden of disease, Inequalities, Physical fitness, Lifestyle and behavior, Health promotion

## Abstract

**Background:**

Low back pain (LBP) is the leading global cause of years lived with disability (YLD), with high body mass index (BMI) recognized as a significant and modifiable risk factor. While prior research has linked high BMI to LBP, comprehensive global assessments of the burden attributable to high BMI are scarce.

**Methods:**

We analyzed data from the Global Burden of Disease (GBD) study (1990–2021) to estimate global and regional YLDs of LBP attributable to high BMI, stratified by age, sex, and socio-demographic index (SDI). The Autoregressive Integrated Moving Average (ARIMA) model projected the future burden to 2050.

**Results:**

From 1990 to 2021, global YLDs of LBP attributable to high BMI nearly doubled, reaching 8.4 million (95 % uncertainty interval: 0.8–17.4), with an age-standardized rate of 97.7 per 100,000. Burdens rose across all regions, with the steepest increases in middle- and lower-SDI areas. Women consistently experienced nearly double the burden compared to men. High-income North America had the largest YLD counts, while South Asia showed the fastest growth. YLDs peaked among individuals aged 40–70, with an emerging burden in the 20–40 age group. Projections suggest disproportionate increases in high- and high-middle-SDI regions by 2050.

**Conclusion:**

The rising burden of LBP attributable to high BMI underscores the urgent need for targeted public health strategies. Integrating BMI management and musculoskeletal health initiatives into healthcare policies could mitigate LBP-related disability and enhance global population health outcomes.

## Highlights

• The Attributable low back pain burden is highest in high socio-demographic index regions but increasing faster in low socio-demographic index regions.
• Age-standardized rates show an inverted V-shaped relationship with socio-demographic index.
• The attributable burden is shifting toward younger age groups.

## 1 Introduction

Low back pain (LBP) has emerged as a critical global health concern, ranking as one of the leading causes of years lived with disability (YLD) and affecting approximately 619 million people worldwide in 2020–a number projected to reach 843 million by 2050 (Ferreira et al., 2023). This increase signals a rising prevalence that will continue to pose a major challenge to global health systems (George et al., 2021). LBP also represents a substantial burden in terms of disability-adjusted life years (DALYs), accounting for 8 % of global DALYs in 2020 (Ferreira et al., 2023). This substantial impact highlights the profound personal and societal effects of LBP, including increased healthcare costs, reduced productivity, and diminished quality of life (Wallwork et al., 2024; Mohd Isa et al., 2022). The rising prevalence of LBP calls for urgent, targeted efforts to identify and manage modifiable risk factors effectively (Ferreira et al., 2023; Chen et al., 2022a).

Among the multiple risk factors for LBP, high body mass index (BMI) has gained prominence due to its modifiable nature and global rise. From 1990 to 2021, global deaths attributable to high BMI rose from 1.5 million to 3.7 million, nearly a 2.5-fold increase, while DALYs associated with high BMI increased from 48 million to 129 million (Zhou et al., 2024). As of 2020, 2.6 billion people worldwide were classified as having excess weight (including overweight and obesity), representing 39 % of the total population, and this proportion is projected to surpass 51 % by 2035 (Nutter et al., 2024). These alarming trends underscore the urgent need for effective prevention strategies to counter the growing public health challenge posed by high BMI.

Excess weight may increase biomechanical stress on spinal structures, a recognized risk factor for LBP development, but also contributes to systemic inflammation, which can interact with psychosocial factors, such as stress and coping mechanisms, in shaping chronic pain trajectories (Mohd Isa et al., 2022; Chiarotto and Koes, 2022). High BMI has been associated with a 1.3-fold increase in premature mortality risk compared to individuals with a healthy weight and a reduction in disease-free life expectancy by three to eight years (Dai et al., 2020). Addressing high BMI, therefore, represents a critical intervention point in reducing LBP burden and enhancing quality of life.

A growing body of research highlights the association between high BMI and LBP (Nitecki et al., 2023; Lucha-López et al., 2023; Liechty et al., 2020; Gilmartin-Thomas et al., 2020; Su et al., 2018). However, despite these findings, few studies have comprehensively evaluated the LBP burden attributed to high BMI on a global scale. This study aims to address this gap by utilizing data from the Global Burden of Disease (GBD) study to examine LBP burden attributable to high BMI across regions, population demographics, and time periods. Through this approach, our findings provide an evidence base for developing targeted BMI management policies and mitigating the substantial global burden of LBP linked to high BMI.

## 2 Methods

### 2.1 Overview

The GBD 2021 offers a robust framework for assessing health loss attributed to a wide range of diseases and risk factors, including those associated with specific conditions (Naghavi et al., 2024; Abbafati et al., 2020). By quantifying health burdens across diverse age, sex, geographic, and socio-demographic groups, GBD enables a comprehensive understanding of the impact of diseases and risk factors through standardized metrics such as DALYs and YLDs. The 2021 iteration presents an expanded assessment of health outcomes, updating burden estimates for 371 diseases and 88 risk factors in 204 countries and territories from 1990 to 2021 (Hay and Collaborators, 2024; Brauer et al., 2024). GBD uniquely provides a systematic and comprehensive update on risk factor exposures and their health impacts, adhering to the Guidelines for Accurate and Transparent Health Estimates Reporting statement (Stevens et al., 2016), thereby ensuring reliability and standardization in global health research.

### 2.2 Definition

In GBD, LBP is defined as pain located in the posterior aspect of the body, specifically from the lower margin of the 12th ribs to the lower gluteal folds. This pain may or may not radiate into one or both lower limbs, and it must persist for at least one day to meet the case definition (Ferreira et al., 2023; Chen et al., 2022b). Relevant diagnostic codes include International Classification of Diseases (ICD)-10 codes M54.3, M54.4, and M54.5, as well as ICD-9 code 724 (Ferreira et al., 2023).

Since low back pain predominantly affects adults, we applied only the adult definition of high BMI. In GBD 2021, high BMI for adults (aged 20 years and older) is defined as a BMI ≥ 25 kg/m^2^ (Brauer et al., 2024, Murray et al., 2020).

### 2.3 Estimation of attributable burden

As there is no reported mortality associated with low back pain, years of life lost (YLLs) are negligible. Thus, DALYs are equivalent to YLDs for low back pain, and we use YLDs as the burden of disease metric in this study.

The YLDs associated with LBP in the GBD framework were estimated utilizing a systematic review of global data sources, including electronic databases, national health surveys. Input data were harmonized by adjusting for sex, age, and variations in case definitions using meta-regression-Bayesian, regularized, trimmed. Prevalence estimates by age, sex, location, and year were generated using the Bayesian meta-regression tool. Severity-specific health states, incorporating presence or absence of leg pain, were defined, each associated with a disability weight. YLDs were calculated by multiplying the prevalence in each health state by its respective disability weight, with adjustments for comorbidity. Detailed methods are available in previous publications (Ferreira et al., 2023; Abbafati et al., 2020; Chen et al., 2022b).

The GBD 2021 study applied a comparative risk assessment (CRA) framework to assess the burden of LBP attributable to high BMI. This analysis followed several core steps: estimating the relative risk of LBP associated with high BMI using a meta-regression approach, determining a theoretical minimum risk exposure level for high BMI (20–25 kg/m^2^) (Murray et al., 2020), and calculating population-attributable fractions to quantify the share of LBP burden linked to this risk factor. DALYs-encompassing YLLs and YLDs-were then derived, with YLDs specifically representing LBP burden due to the absence of mortality data. Additional methodological details on the CRA framework are available in previous publications (Brauer et al., 2024; Murray et al., 2020).

### 2.4 Statistical analysis

We extracted data on YLDs of LBP attributable to high BMI, covering the years 1990–2021, from 204 countries and territories. This analysis was stratified by Socio-demographic Index (SDI), age group, and sex. YLDs and corresponding 95 % uncertainty interval were calculated to express the burden associated with high BMI.

We utilized the estimated annual percentage change (EAPC) as an indicator of long-term trends in age-standardized YLD rates (ASYR), applying a log-linear regression model. Specifically, the logarithm of the annual ASYR was regressed against calendar year x as follows:

$$\ln\left(\text{ASYR}\right)=\alpha +\mathrm{\beta x}+\epsilon .$$

The EAPC and its 95 % confidence interval (CI) were computed as follows:

$$\text{EAPC}=100\times \left(e^{\beta }-1\right)$$

An EAPC lower bound >0 indicated an increasing trend in disease burden, while an upper bound <0 signified a decreasing trend. If 95 % CI contained 0, the trend was considered stable.

To further elucidate the LBP burden attributable to high BMI, we analyzed the YLD burden of LBP alongside the Summary Exposure Values (SEV) of high BMI across 204 countries and territories from 1990 to 2021.

We also examined the association between SDI and attributed LBP burden. The SDI, an index that considers gross domestic product per capita, fertility rate, and education level, ranges from 0 (least developed) to 1 (most developed). Gaussian process regression with a Loess smoother was applied to model the relationship between ASYR and SDI, while the correlation between these variables was assessed using Spearman's rank-order correlation.

To predict future trends in LBP burden attributable to high BMI, we employed an autoregressive integrated moving average (ARIMA) model. The ARIMA model, denoted as $\mathrm{ARIMA}\left(p,d,q\right)$, is widely used for time-series forecasting, where p is the order of autoregressive, d is the order of difference needed to make the series stable, and q is the order of moving average (George, 2015). Parameter (p,d,q)selection was guided by autocorrelation function (ACF) and partial ACF plots, with model selection criteria based on minimizing the Akaike Information Criterion and Bayesian Information Criterion.

All analyses were conducted using R (version 4.2.3), with packages “tidyverse”, “tseries”, “forecast” and “urca”.

### 2.5 Ethics statement

This study uses publicly available or authorized datasets from the GBD project. As the data are anonymized and do not involve direct human subjects research, no additional ethics committee approval is required.

## 3 Results

Globally, LBP burden attributed to high BMI has shown an approximately three-fold increase in YLD, rising from 3.0 million (95 % uncertainty interval: 0.3–6.5) in 1990 to 8.4 million (95 % uncertainty interval: 0.8–17.4) in 2021. The ASYR also increased from 70.2 (7.1–146.5) to 97.7 (9.8–204.0) per 100,000, indicating a rising overall burden (EAPC = 1.14 [95 % CI: 1.11–1.17]). This increasing burden demonstrates marked sex disparities, with women experiencing nearly double the burden compared to men in both YLDs and ASYR (Table 1).

**Table 1:** Global and Regional Years Lived with Disability and Age-Standardized Years Lived with Disability Rates for Low Back Pain Attributable to High Body Mass Index in 1990 and 2021, with Estimated Annual Percentage Change from 1990 to 2021.

Location	YLDs 1990(k)	ASYR 1990 (per 100,000)	YLDs 2021(k)	ASYR 2021 (per 100,000)	EAPC in ASYR
Global	3086.6 (312.6, 6484.4)	70.2 (7.1, 146.5)	8363.8 (840.3, 17,424.8)	97.7 (9.8, 204.0)	1.14 (1.11, 1.17)
Sex					
Male	1014.2 (104.7, 2115.7)	46.6 (4.8, 96.3)	2822.4 (283.7, 5786.4)	67.6 (6.8, 138.9)	1.29 (1.27, 1.31)
Female	2072.3 (207.9, 4368.7)	92.0 (9.2, 193.2)	5541.4 (556.6, 11,636.9)	126.3 (12.6, 266.0)	1.09 (1.05, 1.14)

SDI regions					
High SDI	1218.4 (119.6, 2549.6)	118.8 (11.6, 248.8)	2478.6 (249.2, 5091.8)	161.8 (16.0, 332.6)	1.06 (1.02, 1.09)
High-middle SDI	957.0 (96.3, 2014.4)	91.9 (9.2, 192.0)	2086.2 (212.2, 4263.4)	115.6 (11.6, 238.0)	0.78 (0.72, 0.84)
Middle SDI	547.3 (57.9, 1153.1)	41.9 (4.5, 87.6)	2205.4 (219.7, 4678.3)	79.1 (7.9, 168.2)	2.18 (2.13, 2.22)
Low-middle SDI	276.1 (29.2, 573.9)	35.2 (3.8, 72.7)	1227.1 (122.7, 2554.6)	71.7 (7.2, 148.9)	2.46 (2.41, 2.52)
Low SDI	82.3 (9.1, 165.4)	27.8 (3.1, 55.8)	356.4 (35.0, 730.0)	50.3 (5.1, 103.2)	1.98 (1.92, 2.04)

GBD super regions					
Southeast Asia, East Asia, and Oceania	328.2 (39.1, 679.7)	23.7 (2.9, 48.9)	1460.7 (149.0, 3067.7)	51.8 (5.3, 108.5)	2.87 (2.73, 3.01)
Central Europe, Eastern Europe, and Central Asia	737.6 (71.9, 1547.9)	157.5 (15.3, 329.6)	1168.1 (117.8, 2408.1)	200.9 (19.9, 415.3)	0.85 (0.81, 0.88)
High-income	1272.2 (124.9, 2651.9)	118.2 (11.5, 246.8)	2495.3 (250.6, 5135.7)	163.3 (16.1, 336.1)	1.11 (1.07, 1.15)
Latin America and Caribbean	248.3 (24.3, 524.8)	87.9 (8.7, 184.6)	905.8 (88.7, 1876.5)	141.1 (13.8, 293.1)	1.54 (1.53, 1.56)
North Africa and Middle East	230.9 (22.6, 488.0)	104.9 (10.4, 219.7)	1037.5 (106.6, 2092.7)	179.0 (18.3, 358.3)	1.76 (1.75, 1.78)
South Asia	151.2 (16.6, 302.2)	19.7 (2.2, 39.3)	796.9 (76.4, 1635.3)	46.1 (4.5, 94.4)	3.02 (2.88, 3.15)
Sub-Saharan Africa	118.3 (12.5, 240.8)	43.5 (4.7, 87.9)	499.4 (49.1, 1041.1)	74.9 (7.6, 154.7)	1.74 (1.71, 1.76)

GBD regions					
Andean Latin America	15.5 (1.5, 32.2)	59.9 (5.9, 123.8)	64.8 (6.5, 135.8)	100.2 (10.1, 209.7)	1.72 (1.68, 1.76)
Australasia	35.3 (3.3, 76.1)	157.4 (14.5, 339.8)	91.6 (9.0, 189.4)	227.4 (22.5, 474.3)	1.24 (1.14, 1.33)
Caribbean	18.4 (1.8, 39.4)	62.9 (6.3, 134.7)	50.1 (5.0, 101.7)	96.0 (9.5, 196.5)	1.44 (1.39, 1.49)
Central Asia	60.8 (6.0, 127.2)	119.6 (11.8, 251.4)	140.8 (13.9, 293.3)	151.9 (15.0, 310.8)	0.79 (0.77, 0.81)
Central Europe	248.9 (24.1, 529.5)	170.8 (16.5, 361.7)	383.4 (38.2, 794.8)	217.6 (21.3, 454.1)	0.77 (0.73, 0.81)
Central Latin America	105.2 (10.3, 221.2)	94.1 (9.4, 198.0)	385.1 (38.6, 798.2)	145.4 (14.6, 301.8)	1.42 (1.37, 1.46)
Central Sub-Saharan Africa	8.6 (1.0, 17.7)	28.9 (3.3, 59.4)	52.9 (5.1, 110.7)	67.5 (6.7, 140.7)	2.74 (2.69, 2.79)
East Asia	255.3 (31.2, 520.5)	24.4 (3.0, 49.6)	1109.3 (114.4, 2320.6)	53.2 (5.4, 111.0)	2.9 (2.71, 3.1)
Eastern Europe	427.9 (41.9, 899.4)	157.5 (15.3, 327.7)	643.9 (65.6, 1323.0)	208.2 (20.8, 428.5)	1.01 (0.97, 1.04)
Eastern Sub-Saharan Africa	30.6 (3.4, 62.4)	30.1 (3.4, 60.7)	141.6 (13.9, 294.5)	56.9 (5.8, 116.8)	2.09 (2.07, 2.1)
High-income Asia Pacific	96.5 (11.3, 196.8)	47.6 (5.6, 97.0)	190.3 (21.1, 382.4)	65.1 (7.1, 135.0)	1.02 (0.94, 1.09)
High-income North America	569.6 (53.7, 1213.8)	179.0 (16.9, 382.6)	1131.6 (113.9, 2269.7)	232.5 (23.4, 469.4)	0.95 (0.87, 1.03)
North Africa and Middle East	230.9 (22.6, 488.0)	104.9 (10.4, 219.7)	1037.5 (106.6, 2092.7)	179.0 (18.3, 358.3)	1.76 (1.75, 1.78)
Oceania	2.7 (0.3, 5.7)	64.8 (6.7, 133.5)	8.9 (0.9, 18.5)	83.8 (8.7, 172.4)	0.84 (0.77, 0.9)
South Asia	151.2 (16.6, 302.2)	19.7 (2.2, 39.3)	796.9 (76.4, 1635.3)	46.1 (4.5, 94.4)	3.02 (2.88, 3.15)
Southeast Asia	70.2 (7.6, 142.3)	20.8 (2.3, 41.7)	342.5 (33.4, 723.9)	46.0 (4.5, 95.9)	2.73 (2.65, 2.81)
Southern Latin America	61.9 (6.0, 129.1)	131.3 (12.7, 274.3)	153.2 (15.5, 312.9)	193.1 (19.4, 397.6)	1.28 (1.23, 1.33)
Southern Sub-Saharan Africa	29.9 (3.0, 62.5)	91.8 (9.3, 190.0)	89.5 (9.1, 182.6)	130.1 (13.5, 262.1)	1.15 (1.13, 1.17)
Tropical Latin America	109.2 (10.7, 231.6)	94.5 (9.3, 200.1)	405.7 (38.6, 846.1)	155.9 (14.8, 326.3)	1.62 (1.59, 1.65)
Western Europe	508.9 (50.6, 1070.9)	102.7 (10.1, 216.6)	928.6 (91.1, 1882.8)	141.3 (13.6, 293.9)	1.07 (1.04, 1.09)
Western Sub-Saharan Africa	49.2 (5.1, 100.3)	44.1 (4.6, 89.7)	215.4 (20.8, 460.2)	77.3 (7.7, 163.8)	1.77 (1.73, 1.81)

***Footnote***: GBD: Global Burden of Disease; YLD: Years Lived with Disability. ASYR: Age-Standardized YLD Rates. EAPC: Estimated Annual Percentage Change.

In high-SDI regions, although the LBP burden attributable to high BMI remains substantial, the increase over time has been relatively modest, as evidenced by lower EAPC (1.06 [1.02–1.09]). Conversely, in middle or lower SDI regions, while the baseline burden is comparatively lower, these areas experience more rapid increases, reflected in markedly higher EAPC (Middle SDI: 2.18 [2.13–2.22]; Low-middle-SDI: 2.46 [2.41–2.52]; Low-SDI: 1.98 [1.92–2.04]). Among GBD super-regions, Central Europe, Eastern Europe, and Central Asia had the highest ASYR in 2021, albeit with slower growth, indicated by a relatively low EAPC. South Asia, on the other hand, demonstrated the highest EAPC at 3.02 (2.88–3.15) since 1990, reflecting a more pronounced increase from a lower baseline burden. Notably, high-income regions, especially High-income North America, displayed the largest YLD counts 1.1 million (95 % uncertainty interval: 0.1–2.3), underscoring a significant persistent burden in these developed areas (Table 1).

**Fig. 1:**
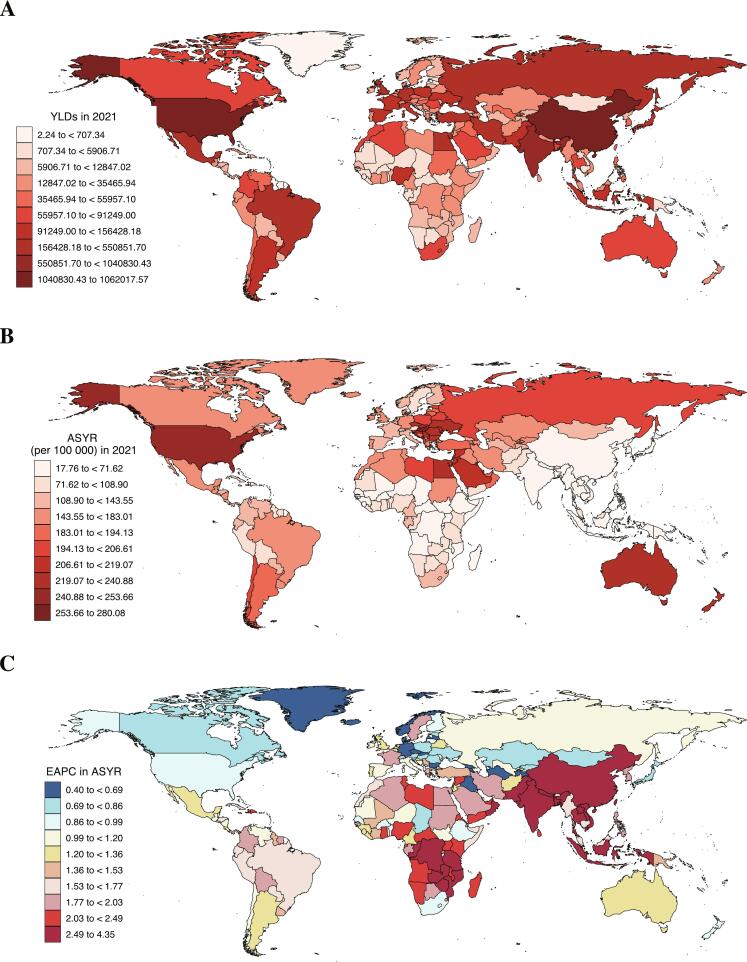
Years Lived with Disability, Age-Standardized Years Lived with Disability Rates, and Estimated Annual Percentage Change in Age-Standardized Years Lived with Disability Rates for Low Back Pain Attributable to High Body Mass Index in 204 Countries and Territories in 2021. ***Footnote***: (A). Years Lived with Disability in 2021; (B). Age-Standardized Years Lived with Disability Rates (per 100,000) in 2021; (C). Estimated Annual Percentage Change (%). YLD: Years Lived with Disability. ASYR: Age-Standardized YLD Rates. EAPC: Estimated Annual Percentage Change.

Among 204 countries and territories, the majority exhibited a high LBP burden attributable to high BMI. Age-standardized results showed a significant relative decrease in burden rank for some countries or regions (e.g., China, India, especially in South Asia), but with substantial EAPC increases. Conversely, the ASYR has remained persistently high in countries like the United States, Australia, and regions in North Africa and the Middle East (Fig. 1, and **Table S1**).

**Fig. 2:**
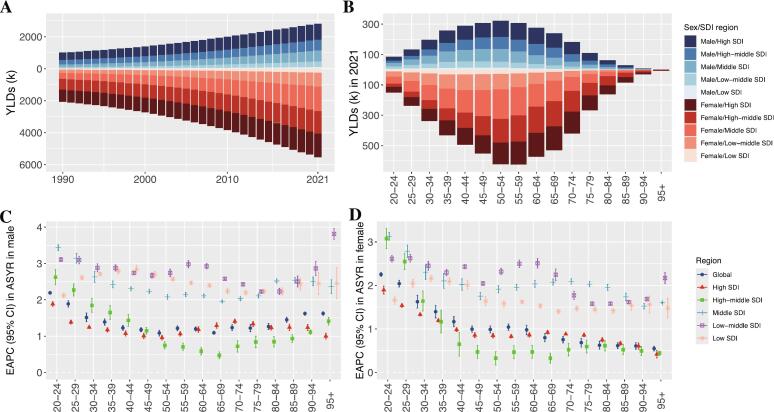
Years Lived with Disability for Low Back Pain Attributable to High Body Mass Index in Global and Five Socio-Demographic Index Regions from 1990 to 2021, and Across Five-Year Age Groups, with Estimated Annual Percentage Change in Age-Standardized Years Lived with Disability Rates for Males and Females. ***Footnote***: (A). Years Lived with Disability (thousands) from 1990 to 2021 in Five Socio-Demographic Index Regions, Males and Females; (B). Years Lived with Disability (thousands) in Five-Year Age Groups in 2021; (C). Estimated Annual Percentage Change and 95 % Confidence Interval in Age-Standardized Years Lived with Disability Rates in Males; (D). Estimated Annual Percentage Change and 95 % Confidence Interval in Age-Standardized Years Lived with Disability Rates in Females. YLD: Years Lived with Disability. ASYR: Age-Standardized YLD Rates. EAPC: Estimated Annual Percentage Change. SDI: Socio-Demographic Index.

From 1990 to 2021, the LBP burden attributable to high BMI has consistently increased, particularly in regions with moderate to high development levels and among women (Fig. 2A). The age distribution of disease burden forms an elliptical pattern, with YLDs concentrated among individuals aged 40 to 70, regardless of sex (Fig. 2B). ASYR trends show an upward trajectory across all age groups (EAPC >0), with marked increases in the 20–40 age bracket for both men and women. Additionally, middle- and lower-SDI regions exhibited significant increases in burden across all age categories compared to other areas (Fig. 2C **&D**). Detailed EAPC values can be found in **Table S2.**

Our results (**Fig.S1** and **Fig.S2**) revealed that the ASYRs for LBP were particularly high in high- and high-middle-SDI regions, including the United States, Australia, Eastern Europe, and Russia. Over the period from 1990 to 2021, standardized results showed a plateauing or declining trend in LBP burden. In contrast, the SEV rates of high BMI remained elevated in these regions, with a concerning trend of continuous increase across all SDI levels, regardless of sex.

**Fig. 3:**
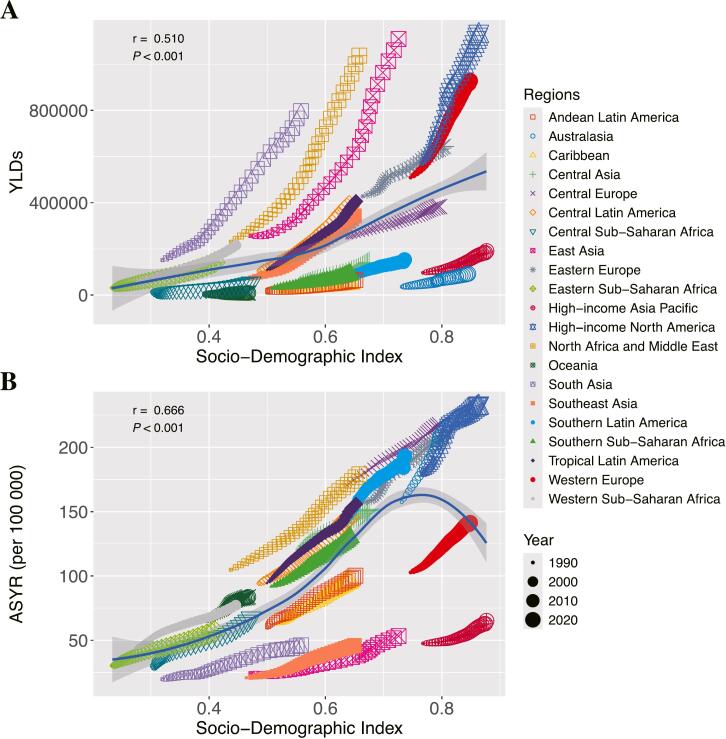
Correlation Analysis of Years Lived with Disability and Age-Standardized Years Lived with Disability Rates for Low Back Pain Attributable to High Body Mass Index with Socio-Demographic Index Across 21 Global Burden of Disease Regions, 1990–2021. ***Footnote***: (A). Scatter Plot Between Socio-Demographic Index and Years Lived with Disability from 1990 to 2021; (B). Scatter Plot Between Socio-Demographic Index and Age-Standardized Years Lived with Disability Rates (per 100,000) from 1990 to 2021. YLD: Years Lived with Disability. ASYR: Age-Standardized YLD Rates.

**Fig. 4:**
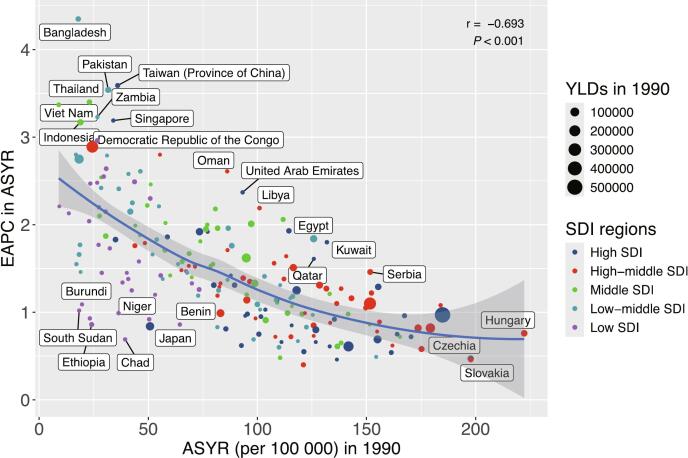
Correlation Analysis Between the Estimated Annual Percentage Change in Age-Standardized Years Lived with Disability Rates for Low Back Pain Attributable to High Body Mass Index and the Initial Age-Standardized Years Lived with Disability Rates in 1990 Across 204 Countries and Territories. YLD: Years Lived with Disability. ASYR: Age-Standardized YLD Rates. EAPC: Estimated Annual Percentage Change. SDI: Socio-Demographic Index.

A strong positive correlation was observed between YLDs of LBP attributable to high BMI and the SDI (Fig. 3A). For ASYR, a positive correlation was evident in areas where SDI was below approximately 0.75. However, as SDI surpassed 0.75, ASYR began to decrease with rising SDI, resulting in an asymmetric inverted V-shape relationship (Fig. 3B). EAPC values were significantly negatively correlated with ASYR in 1990 (Fig. 4).

**Fig. 5:**
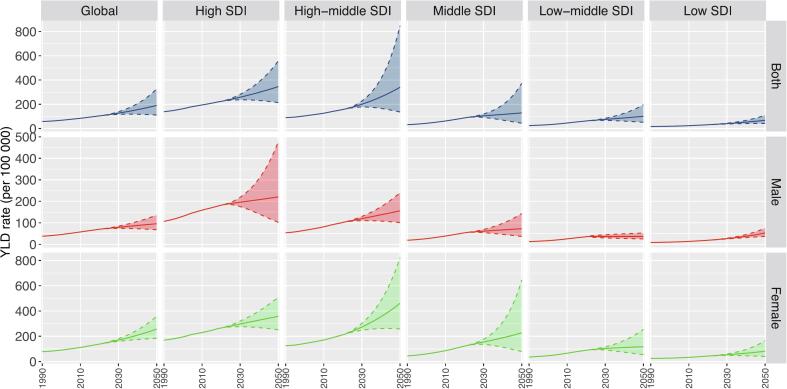
Time Trend of Years Lived with Disability Rates (per 100,000) for Low Back Pain Attributable to High Body Mass Index in Global and Five Socio-Demographic Index Regions for Both Males and Females, 1990–2021, with Projections from 2022 to 2050. YLD: Years Lived with Disability. SDI: Socio-Demographic Index.

From a temporal perspective, the global and regional LBP burden attributed to high BMI, in both all-ages YLD rates and ASYR, exhibited upward trends from 1990 to 2021 across all SDI levels (Fig. 5). Predictive models suggest that if unmitigated, the global YLD rate attributable to high BMI-related LBP could reach 190.5 (111.1–326.6) per 100,000 by 2050, with high- and high-middle SDI regions bearing a disproportionately high burden (**Table S3**).

## 4 Discussion

Our study provides a comprehensive assessment of the global burden of LBP attributable to high BMI, measured in YLDs. Overall, the burden of LBP attributed to high BMI is a growing concern globally, with the 2021 burden nearly tripling since 1990. This increase highlights significant geographic and sex disparities, underscoring the urgent need for targeted, effective interventions addressing this modifiable risk factor to control the LBP burden.

Notable regional differences in YLDs attributable to high BMI reveal distinct patterns. High-income countries or region–such as the United States, Australia, and New Zealand–tend to exhibit a markedly higher LBP burden compared to other areas. This pattern diverges from the common perception that disease burdens are more pronounced in lower-income populations. Such differences may be explained by the greater prevalence of sedentary lifestyles, physical inactivity, smoking, and sedentary or repetitive labor in high-income countries, all of which are risk factors for LBP (Ferreira et al., 2023; Dai et al., 2020). Moreover, data on LBP prevalence in low- and middle-income countries are limited, constraining our understanding of the burden in these regions (Ferreira et al., 2023). Despite the high LBP burden in developed nations, our findings suggest that the rate of burden increase is slower in these regions, likely due to more resources and policies supporting obesity prevention and management. In contrast, less developed countries, while currently showing a lower overall burden, have experienced a much higher growth rate in LBP burden attributable to high BMI since 1990, as indicated by elevated EAPC values. This could be attributed to resource limitations in promoting healthier diets and lifestyles, contributing to rising obesity rates in these low-SDI regions (Dai et al., 2020).

The persistent increase in LBP burden attributable to high BMI can be explained by the rising SEV rates of high BMI, which have been continuously increasing across all SDI levels from 1990 to 2021. This suggests that the growing prevalence of high BMI is a major driving factor behind the sustained increase in LBP burden. The overlap between high BMI exposure and regions with high LBP ASYR burdens underscores the compounded impact of these factors, contributing to disproportionately high attributable burdens in specific areas.

Sex disparities were also evident, with women experiencing a significantly higher LBP burden due to high BMI than men. This association may be influenced by a combination of biopsychosocial factors, such as differences in the susceptibility to obesity, structure of the pelvis and lumbar spine, pain perception, and coping strategies (Keogh and Herdenfeldt, 2002; El-Shormilisy et al., 2015). Additionally, women may be more inclined to seek medical care for LBP, resulting in more accurate prevalence data (Ferreira et al., 2023).

When analyzing the age-standardized YLD rate, we found that in certain countries–such as China–the ASYR was substantially lower than in other nations (Fig. 1). Given China's significant aging population, this suggests that population aging likely contributes considerably to the LBP burden. Additionally, the burden of LBP attributable to high BMI predominantly impacts individuals in middle age, who constitute a critical segment of the workforce. The associated disability can lead to substantial losses in the available labor force. Alarmingly, we also observed a trend toward younger onset, with a higher EAPC in individuals under 40, signaling an urgent need for life-course chronic disease management strategies.

Our findings corroborate the observed regional disparities by showing a significant positive correlation between SDI and YLDs. For ASYR, the inverted V-shaped pattern suggests that as SDI increases beyond a certain threshold, the burden of LBP attributable to high BMI may decrease in relation to population characteristics, particularly in high-SDI regions. This pattern aligns with our stratified analysis based on World Bank income levels (**Fig.S3**), which further confirmed that the bulk of the LBP burden attributable to high BMI is concentrated in high- and upper-middle-income regions. This emphasizes the pressing need for targeted interventions to reduce the LBP burden, particularly in developed regions where the prevalence remains highest.

This study has several limitations. First, BMI data partly relies on self-reports, which may introduce measurement bias despite correction methods. BMI as an indicator does not account for individual differences in body composition or fat distribution, potentially leading to inaccuracies across diverse populations. Additionally, uniform BMI thresholds may not reflect variations in health risk across global populations. Meanwhile, the GBD database provides High BMI (≥ 25 kg/m^2^) data only at the country level, limiting our ability to examine a dose-response relationship with more refined BMI categories. For LBP data, diverse sources and inconsistent case definitions increase uncertainty, and limited covariates constrain our ability to assess LBP's relationship with other risk factors. Furthermore, data scarcity from low- and middle-income countries limits representativeness, while model-based estimates for certain regions may introduce bias. Finally, the study focuses only on non-fatal LBP burden, without capturing mortality or fully assessing the impact of risk factor prevention strategies.

## 5 Conclusion

Our study highlights the significant burden of LBP attributable to high BMI, underscoring the critical need for comprehensive strategies targeting both BMI management and LBP prevention on a global scale. With the prevalence of high BMI rising across all age groups and countries, its contribution to musculoskeletal and other chronic diseases is expected to increase further, intensifying the strain on healthcare systems and reducing quality of life worldwide. Given that LBP remains the leading cause of disability globally, our findings reinforce the importance of integrating BMI management into public health initiatives aimed at mitigating LBP. Interventions that focus on lifestyle modifications, weight control, and targeted musculoskeletal health strategies can substantially reduce the burden of both conditions. Ultimately, coordinated public health policies that address BMI as a modifiable risk factor could lead to meaningful reductions in LBP prevalence and associated disabilities, improving overall population health and easing healthcare costs globally.

## Funding

This study was supported by the 10.13039/501100012166National Key Research and Development Program of China (Grant No. 2022YFC3703000) and the CAMS Innovation Fund for Medical Sciences (Grant No. 2022-I2M-JB-003).

## CRediT authorship contribution statement

**Fan Wang:** Writing – review & editing, Writing – original draft, Validation, Methodology, Data curation, Conceptualization. **Yisen Yang:** Writing – review & editing, Writing – original draft, Visualization, Software, Methodology, Formal analysis, Conceptualization. **Jing Xu:** Validation. **Meiduo Zhao:** Validation. **Hongwei Ma:** Writing – review & editing, Writing – original draft, Validation. **Qun Xu:** Writing – review & editing, Writing – original draft, Supervision, Funding acquisition.

## Declaration of competing interest

There is no known competing financial interests or personal relationships that could have appeared to influence the work reported in this paper.

## Data Availability

The datasets generated during and/or analyzed during the current study are available from https://ghdx.healthdata.org/gbd-2021. The principal authors take full responsibility for the analyses, interpretation, and the conduct of the research.

## Appendix A Supplementary data

Supplementary material Image 1
