# Supplementary material for: The shifting global landscape of low Back pain attributable to high body mass index: Burden, growth, and inequalities
Source: Prev Med Rep. 2025 Mar 12;53:103031. doi: 10.1016/j.pmedr.2025.103031 (PMC11964669; doi:10.1016/j.pmedr.2025.103031)
Supplement: Supplementary file 1 — Supplementary material [file mmc1.docx]

**The Shifting Global Landscape of Low Back Pain Attributable to High Body Mass Index: Burden, Growth, and Inequalities**

Fan Wang^a,b,1^, Yisen Yang^b,c,1^, Jing Xu^b,c^, Meiduo Zhao^b,c^, Hongwei Ma^d^*, Qun Xu^b,e,^*

^a^ School of Population Medicine and Public Health, Chinese Academy of Medical Sciences & Peking Union Medical College, Beijing 100730, China;

^b^ Department of Epidemiology and Biostatistics, Institute of Basic Medical Sciences Chinese Academy of Medical Sciences, School of Basic Medicine Peking Union Medical College, Beijing 100005, China;

^c^ Center of Environmental and Health Sciences, Chinese Academy of Medical Sciences, Peking Union Medical College, Beijing 100005, China;

^d^ Department of Rehabilitation Medicine & Department of Pain Medicine, Peking University International Hospital & PKUCare Rehabilitation Hospital, Beijing 102206, China

^e^ Center for Rare Diseases, State Key Laboratory of Complex, Severe, and Rare Diseases, Peking Union Medical College Hospital, Chinese Academy of Medical Sciences, Beijing, 100730, China

^1^ Contributed equally, therefore they are co-ﬁrst authors.

* Correspondence to:

Hongwei Ma, Ph.D.

Department of Rehabilitation Medicine & Department of Pain Medicine

Peking University International Hospital & PKUCare Rehabilitation Hospital

Beijing 102206, China

Tel.: +86 10 69006816

Fax: +86 10 69006816

E-mail: [MAHONGWEI138@pkucare.com](mailto:MAHONGWEI138@pkucare.com)

Qun Xu, Ph.D.

Center for Rare Diseases, State Key Laboratory of Complex, Severe, and Rare Diseases, Peking Union Medical College Hospital, Chinese Academy of Medical Sciences

Beijing 100730, China

Tel.: +86 10 69154667

Fax: +86 10 69154667

E-mail: [xuqun@ibms.cams.cn](mailto:xuqun@ibms.cams.cn)

**Contents of Supplemental Materials**

[Supplementary Figure S1. Years Lived with Disability and Age-Standardized Years Lived with Disability Rates for Low Back Pain in 204 Countries and Territories in 2021, and Time Trends in Global and Five Socio-Demographic Index Regions from 1990 to 2021, for Both Males and Females. 4](#_Toc191927572)

[Supplementary Figure S2. Summary Exposure Value of Age-Standardized Rates of High Body Mass Index Across 204 Countries and Territories in 2021, with Time Trends in Global and Five Socio-Demographic Index Regions from 1990 to 2021, for Males and Females. 5](#_Toc191927573)

[Supplementary Figure S3. Years Lived with Disability and Age-Standardized Years Lived with Disability Rates for Low Back Pain Attributable to High Body Mass Index in Global and Four World Bank Income Regions from 1990 to 2021, and Across Five-Year Age Groups. 6](#_Toc191927574)

[Supplementary Table S1. Years Lived with Disability and Age-Standardized Years Lived with Disability Rates for Low Back Pain Attributable to High Body Mass Index in 204 Countries and Territories in 1990 and 2021, with Estimated Annual Percentage Change from 1990 to 2021. 7](#_Toc191927575)

[Supplementary Table S2. Estimated Annual Percentage Change in Age-Standardized Years Lived with Disability Rates for Low Back Pain Attributable to High Body Mass Index by Five-Year Age Groups in Global and Five Socio-Demographic Index Regions for Both Males and Females, 1990–2021. 16](#_Toc191927576)

[Supplementary Table S3. Predicted Years Lived with Disability Rates (per 100,000) for Low Back Pain Attributable to High Body Mass Index in Global and Five Socio-Demographic Index Regions for Both Males and Females from 2022 to 2050. 24](#_Toc191927577)


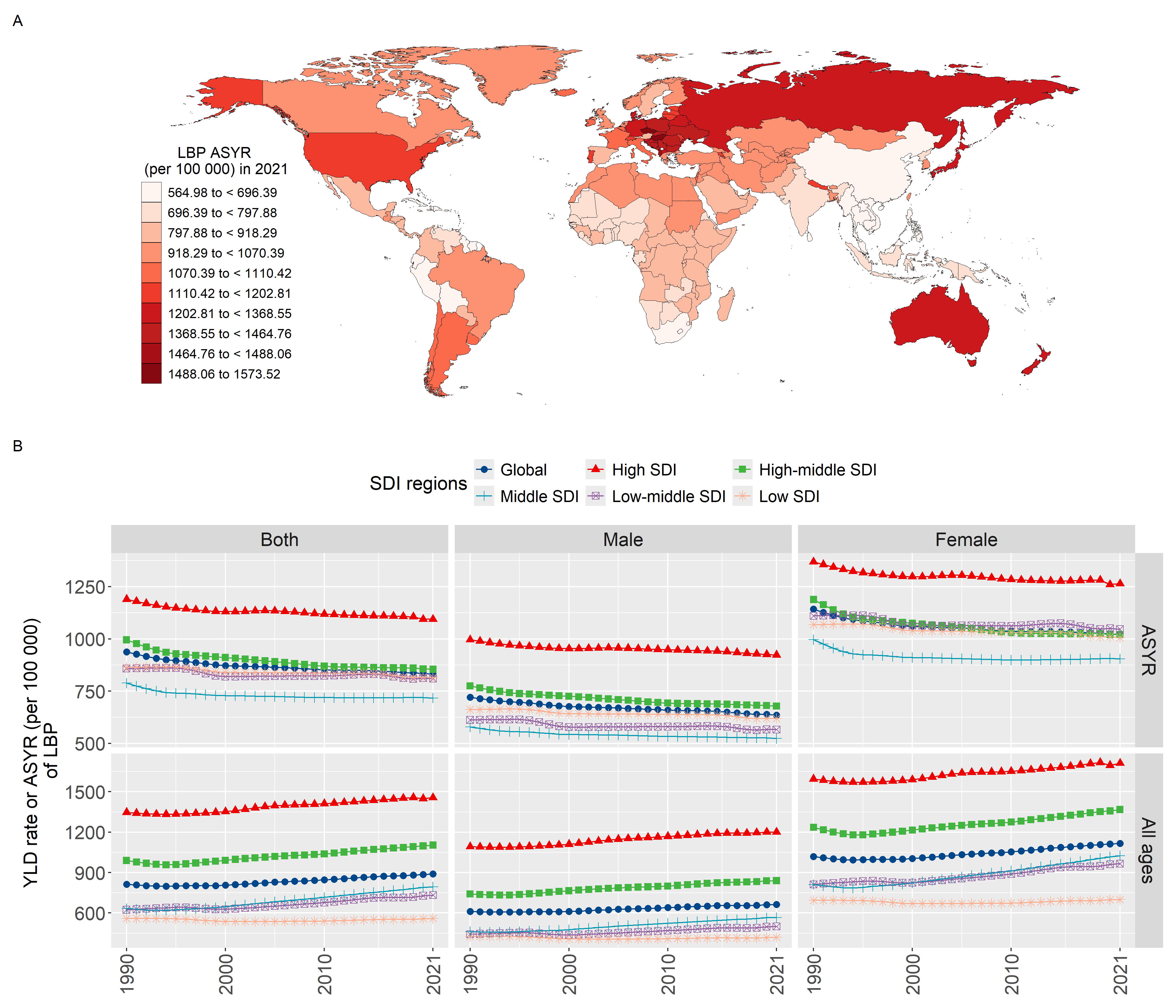


## Supplementary Figure S1. Years Lived with Disability and Age-Standardized Years Lived with Disability Rates for Low Back Pain in 204 Countries and Territories in 2021, and Time Trends in Global and Five Socio-Demographic Index Regions from 1990 to 2021, for Both Males and Females.

***Footnote***: LBP: Low back pain; ASYR： Age-standardized YLD rates；SDI： Socio-demographic index; YLD: Years Lived with Disability.


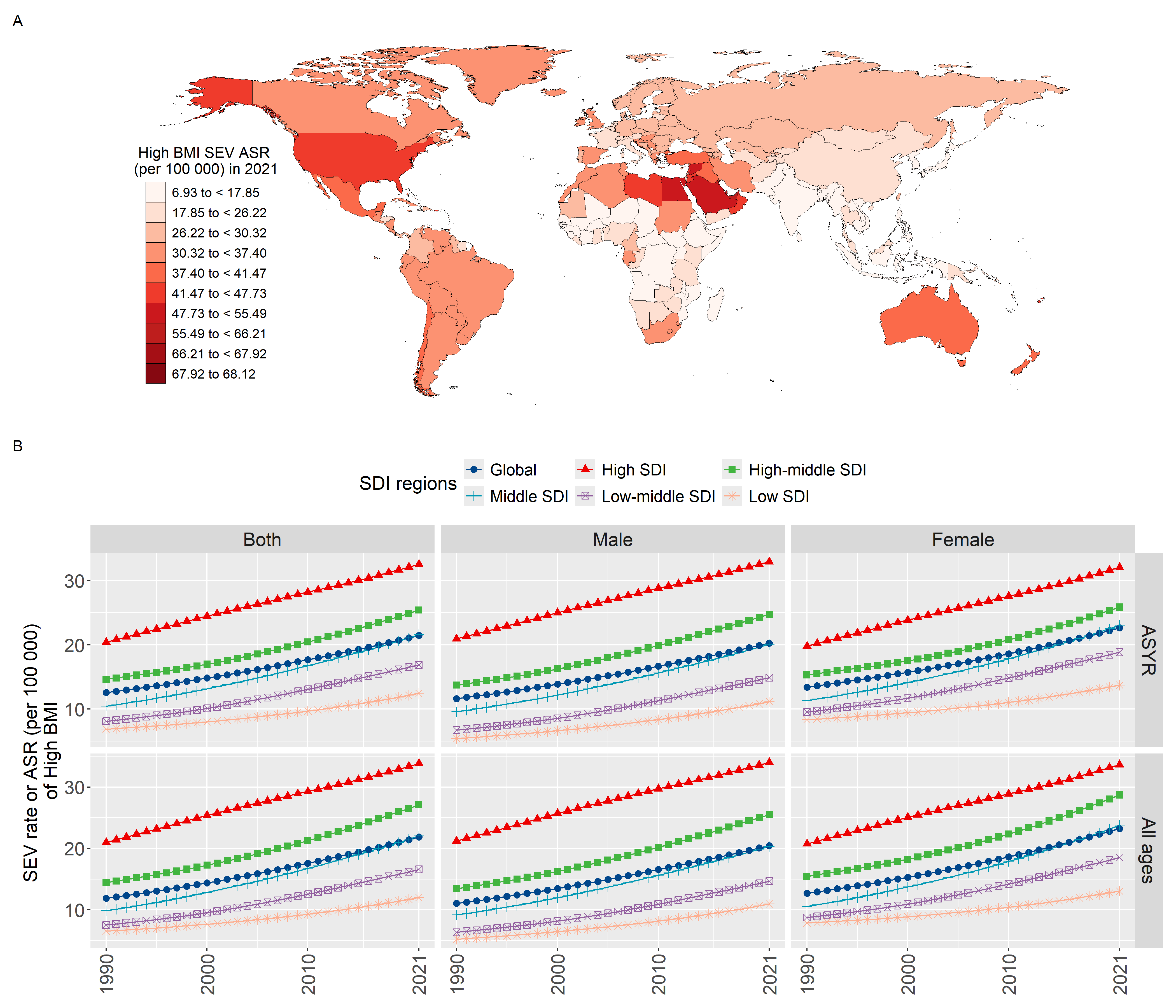


## Supplementary Figure S2. Summary Exposure Value of Age-Standardized Rates of High Body Mass Index Across 204 Countries and Territories in 2021, with Time Trends in Global and Five Socio-Demographic Index Regions from 1990 to 2021, for Males and Females.

***Footnote***: BMI: Body mass index; SEV: Summary exposure values; ASR: Age-standardized rate; SDI: Socio-demographic index.


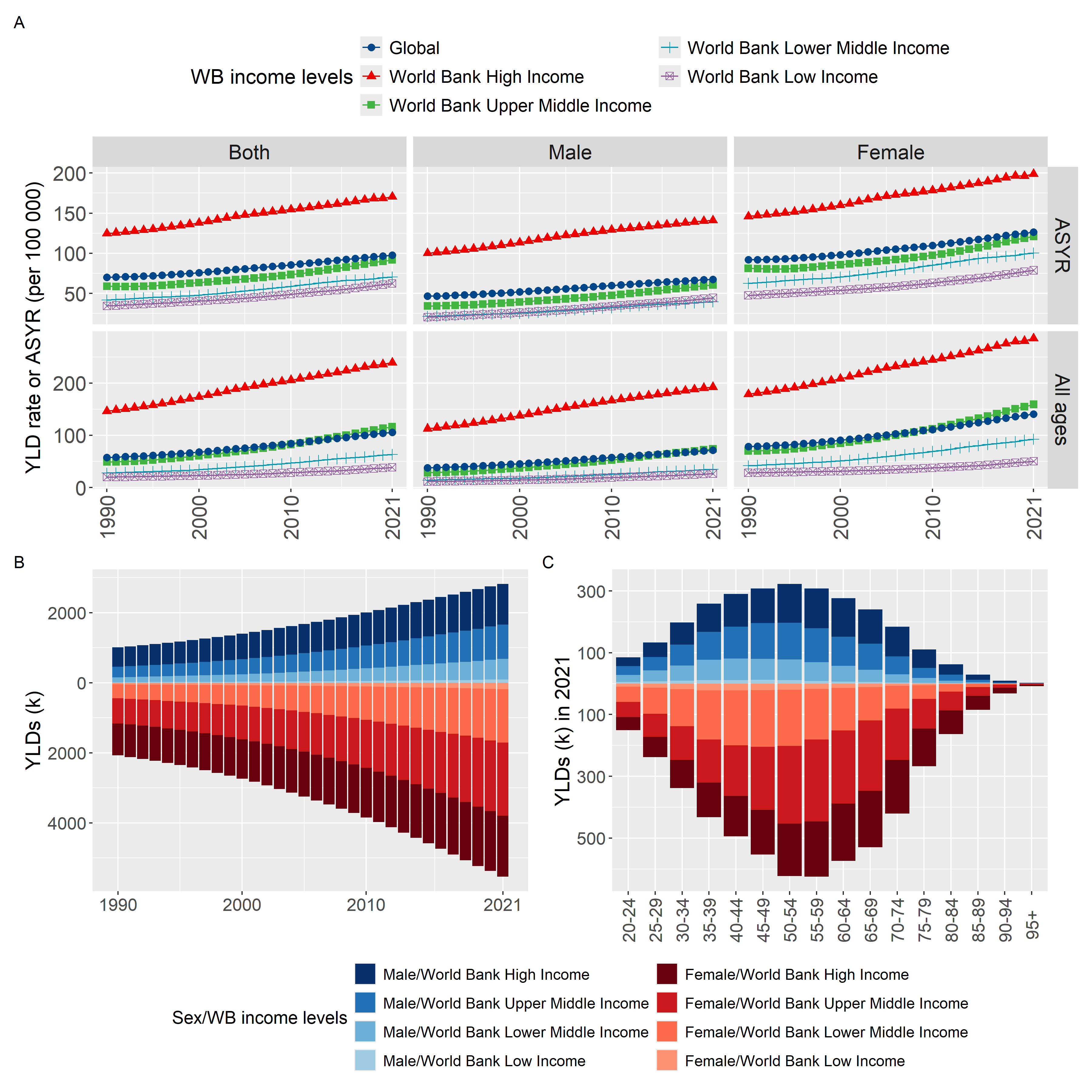


## Supplementary Figure S3. Years Lived with Disability and Age-Standardized Years Lived with Disability Rates for Low Back Pain Attributable to High Body Mass Index in Global and Four World Bank Income Regions from 1990 to 2021, and Across Five-Year Age Groups.

***Footnote***: WB: World bank; YLD: Years lived with disability; ASYR: Age-standardized YLD rates; YLDs: Years lived with disability.

##

## Supplementary Table S1. Years Lived with Disability and Age-Standardized Years Lived with Disability Rates for Low Back Pain Attributable to High Body Mass Index in 204 Countries and Territories in 1990 and 2021, with Estimated Annual Percentage Change from 1990 to 2021.

| Location | YLDs 1990(k) | ASYR 1990  (per 100 000) | YLDs 2021(k) | ASYR 2021  (per 100 000) | EAPC in ASYR |
| --- | --- | --- | --- | --- | --- |
| Afghanistan | 5.2 (0.5, 10.8) | 73.3 (7.1, 151.5) | 18.8 (1.7, 39.1) | 107.7 (10.3, 223.7) | 1.26 (1.22, 1.31) |
| Albania | 3.5 (0.3, 7.2) | 141.6 (13.6, 299.4) | 7.3 (0.7, 14.9) | 194.5 (19.2, 397.2) | 0.98 (0.95, 1.02) |
| Algeria | 12.7 (1.3, 26.7) | 81.8 (8.3, 171.0) | 64.7 (6.6, 135.0) | 150.8 (15.2, 311.6) | 1.96 (1.94, 1.99) |
| American Samoa | 0.1 (0.0, 0.1) | 161.9 (17.2, 325.2) | 0.1 (0.0, 0.2) | 194.4 (21.7, 376.7) | 0.62 (0.58, 0.65) |
| Andorra | 0.1 (0.0, 0.1) | 94.4 (9.4, 196.2) | 0.2 (0.0, 0.3) | 124.3 (12.0, 260.2) | 0.96 (0.90, 1.02) |
| Angola | 1.6 (0.2, 3.3) | 28.4 (3.3, 57.7) | 11.0 (1.1, 22.5) | 61.2 (6.2, 126.0) | 2.42 (2.36, 2.48) |
| Antigua and Barbuda | 0.0 (0.0, 0.1) | 68.6 (6.7, 145.9) | 0.1 (0.0, 0.3) | 109.0 (10.8, 232.5) | 1.53 (1.49, 1.56) |
| Argentina | 41.3 (4.1, 87.9) | 128.5 (12.7, 272.6) | 98.2 (10.0, 201.3) | 189.4 (19.2, 390.2) | 1.31 (1.26, 1.37) |
| Armenia | 4.1 (0.4, 8.5) | 138.4 (13.5, 285.7) | 6.7 (0.7, 13.9) | 168.6 (16.8, 349.8) | 0.65 (0.62, 0.67) |
| Australia | 29.1 (2.7, 63.5) | 155.4 (14.2, 339.5) | 77.4 (7.7, 159.8) | 229.5 (22.9, 478.5) | 1.29 (1.18, 1.40) |
| Austria | 8.8 (0.9, 18.2) | 85.5 (8.7, 178.7) | 14.6 (1.4, 29.9) | 104.8 (10.3, 215.3) | 0.81 (0.74, 0.89) |
| Azerbaijan | 6.7 (0.7, 14.0) | 120.4 (12.0, 252.8) | 18.9 (1.9, 39.9) | 160.8 (16.1, 338.1) | 0.98 (0.95, 1.01) |
| Bahamas | 0.2 (0.0, 0.4) | 82.6 (8.1, 178.2) | 0.5 (0.1, 1.1) | 124.1 (12.8, 251.5) | 1.29 (1.26, 1.33) |
| Bahrain | 0.4 (0.0, 1.0) | 113.2 (11.0, 241.5) | 3.1 (0.3, 6.3) | 189.5 (20.4, 380.1) | 1.64 (1.62, 1.67) |
| Bangladesh | 11.7 (1.4, 23.4) | 17.9 (2.1, 35.5) | 89.8 (8.8, 187.8) | 56.1 (5.6, 117.1) | 4.35 (4.14, 4.56) |
| Barbados | 0.2 (0.0, 0.5) | 83.1 (8.2, 177.0) | 0.5 (0.1, 1.1) | 127.0 (12.7, 261.8) | 1.33 (1.27, 1.39) |
| Belarus | 16.5 (1.6, 34.7) | 131.6 (13.0, 276.1) | 27.6 (2.7, 56.6) | 194.2 (19.0, 396.4) | 1.27 (1.24, 1.31) |
| Belgium | 11.8 (1.2, 24.7) | 89.5 (9.0, 189.0) | 22.3 (2.2, 46.7) | 134.7 (13.0, 283.1) | 1.31 (1.24, 1.38) |
| Belize | 0.1 (0.0, 0.2) | 101.2 (9.8, 216.2) | 0.5 (0.1, 1.1) | 138.1 (14.7, 285.5) | 1.03 (0.99, 1.08) |
| Benin | 1.6 (0.2, 3.3) | 64.5 (6.5, 134.6) | 6.4 (0.6, 13.5) | 86.1 (8.3, 180.5) | 0.86 (0.83, 0.90) |
| Bermuda | 0.1 (0.0, 0.1) | 87.8 (9.0, 178.4) | 0.1 (0.0, 0.3) | 136.3 (13.7, 274.9) | 1.42 (1.37, 1.47) |
| Bhutan | 0.2 (0.0, 0.5) | 63.7 (6.8, 131.2) | 0.7 (0.1, 1.5) | 99.1 (9.7, 205.7) | 1.55 (1.51, 1.59) |
| Bolivia (Plurinational State of) | 2.2 (0.2, 4.7) | 53.9 (5.5, 111.7) | 10.3 (1.0, 22.0) | 95.1 (9.4, 202.5) | 1.83 (1.73, 1.93) |
| Bosnia and Herzegovina | 6.3 (0.6, 13.4) | 136.4 (13.1, 290.9) | 9.8 (1.0, 20.4) | 194.1 (18.8, 407.5) | 1.10 (1.04, 1.15) |
| Botswana | 0.4 (0.0, 0.8) | 57.9 (5.9, 119.7) | 2.1 (0.2, 4.4) | 108.9 (10.1, 220.3) | 2.02 (1.94, 2.10) |
| Brazil | 107.0 (10.5, 227.0) | 94.9 (9.4, 200.7) | 397.2 (37.8, 828.1) | 156.7 (14.9, 327.9) | 1.62 (1.59, 1.65) |
| Brunei Darussalam | 0.1 (0.0, 0.2) | 55.3 (5.8, 114.1) | 0.6 (0.1, 1.4) | 125.4 (12.1, 264.5) | 2.80 (2.57, 3.04) |
| Bulgaria | 20.1 (2.0, 43.4) | 175.2 (17.2, 378.8) | 22.8 (2.2, 47.2) | 208.9 (20.3, 435.8) | 0.58 (0.55, 0.60) |
| Burkina Faso | 1.3 (0.1, 2.7) | 23.2 (2.8, 48.5) | 4.2 (0.5, 8.9) | 31.7 (3.5, 65.5) | 0.93 (0.87, 0.98) |
| Burundi | 0.6 (0.1, 1.2) | 19.6 (2.2, 39.7) | 2.1 (0.2, 4.2) | 28.7 (2.9, 58.8) | 1.09 (0.96, 1.21) |
| Cabo Verde | 0.1 (0.0, 0.2) | 51.9 (5.1, 107.0) | 0.5 (0.0, 1.0) | 88.0 (8.9, 186.8) | 1.66 (1.63, 1.69) |
| Cambodia | 1.0 (0.1, 2.2) | 17.1 (2.0, 36.5) | 4.1 (0.4, 8.7) | 27.2 (2.9, 56.7) | 1.55 (1.51, 1.59) |
| Cameroon | 4.8 (0.5, 10.3) | 82.2 (8.0, 174.8) | 22.7 (2.2, 47.9) | 122.8 (12.5, 253.1) | 1.25 (1.18, 1.31) |
| Canada | 39.9 (3.8, 84.5) | 126.8 (12.1, 269.3) | 83.2 (8.5, 173.3) | 159.5 (15.8, 336.3) | 0.80 (0.77, 0.84) |
| Central African Republic | 0.4 (0.0, 0.9) | 26.6 (2.9, 55.5) | 1.9 (0.2, 4.0) | 56.4 (5.6, 118.7) | 2.48 (2.39, 2.57) |
| Chad | 1.3 (0.1, 2.7) | 39.4 (3.9, 82.2) | 3.9 (0.4, 8.5) | 49.5 (4.6, 107.0) | 0.69 (0.66, 0.72) |
| Chile | 16.6 (1.5, 36.3) | 143.1 (13.4, 308.5) | 47.6 (4.8, 97.5) | 205.4 (20.3, 423.3) | 1.16 (1.11, 1.21) |
| China | 245.2 (30.0, 500.1) | 24.3 (3.0, 49.3) | 1062.0 (110.0, 2225.1) | 52.7 (5.4, 110.1) | 2.89 (2.69, 3.10) |
| Colombia | 17.8 (1.8, 37.5) | 76.4 (7.7, 160.7) | 73.6 (7.0, 155.2) | 134.4 (12.8, 283.2) | 1.95 (1.89, 2.00) |
| Comoros | 0.1 (0.0, 0.2) | 31.1 (3.3, 62.6) | 0.5 (0.0, 0.9) | 73.2 (7.1, 153.5) | 2.79 (2.76, 2.81) |
| Congo | 0.6 (0.1, 1.2) | 40.9 (4.5, 84.6) | 3.7 (0.3, 7.9) | 91.7 (8.7, 192.2) | 2.58 (2.47, 2.69) |
| Cook Islands | 0.0 (0.0, 0.0) | 154.9 (16.1, 314.4) | 0.0 (0.0, 0.1) | 193.8 (21.9, 375.5) | 0.78 (0.76, 0.80) |
| Costa Rica | 2.1 (0.2, 4.6) | 95.1 (9.4, 202.9) | 7.4 (0.7, 15.5) | 136.8 (13.5, 284.7) | 1.20 (1.18, 1.21) |
| Croatia | 9.2 (0.9, 19.5) | 152.3 (14.8, 318.9) | 14.4 (1.4, 30.3) | 218.5 (21.6, 461.7) | 1.22 (1.11, 1.34) |
| Cuba | 7.1 (0.7, 14.7) | 65.5 (6.4, 137.2) | 16.0 (1.5, 32.8) | 101.2 (9.5, 206.8) | 1.58 (1.52, 1.64) |
| Cyprus | 0.6 (0.1, 1.3) | 77.2 (8.3, 161.6) | 2.6 (0.3, 5.4) | 139.1 (13.7, 294.9) | 1.92 (1.82, 2.02) |
| Czechia | 25.3 (2.4, 53.0) | 197.7 (18.6, 415.6) | 38.0 (3.8, 79.5) | 226.9 (22.1, 476.5) | 0.47 (0.45, 0.49) |
| Côte d’Ivoire | 3.6 (0.4, 7.1) | 55.7 (5.8, 112.3) | 15.1 (1.4, 32.2) | 86.3 (8.4, 184.3) | 1.42 (1.35, 1.49) |
| Democratic People’s Republic of Korea | 3.4 (0.5, 6.9) | 18.7 (2.5, 38.0) | 10.7 (1.2, 22.5) | 33.4 (3.8, 69.3) | 1.77 (1.60, 1.95) |
| Democratic Republic of the Congo | 5.4 (0.6, 11.1) | 26.6 (3.0, 54.5) | 33.5 (3.2, 71.5) | 66.1 (6.5, 141.3) | 2.96 (2.89, 3.03) |
| Denmark | 8.2 (0.8, 16.8) | 121.3 (11.3, 252.1) | 13.0 (1.2, 27.2) | 154.9 (14.3, 328.3) | 0.55 (0.42, 0.68) |
| Djibouti | 0.0 (0.0, 0.1) | 19.0 (2.3, 38.4) | 0.3 (0.0, 0.6) | 30.5 (3.2, 62.5) | 1.46 (1.39, 1.53) |
| Dominica | 0.1 (0.0, 0.1) | 99.1 (9.8, 204.9) | 0.1 (0.0, 0.2) | 133.3 (14.1, 273.9) | 0.93 (0.90, 0.95) |
| Dominican Republic | 2.4 (0.2, 5.2) | 49.5 (5.0, 104.1) | 10.7 (1.0, 22.9) | 97.7 (9.5, 208.6) | 2.22 (2.14, 2.29) |
| Ecuador | 4.7 (0.5, 10.3) | 68.7 (6.8, 150.2) | 19.8 (2.1, 41.1) | 113.3 (11.9, 234.3) | 1.67 (1.56, 1.77) |
| Egypt | 46.8 (4.6, 98.6) | 125.8 (12.4, 262.9) | 187.7 (20.2, 368.2) | 219.2 (23.5, 428.2) | 1.84 (1.79, 1.90) |
| El Salvador | 3.7 (0.4, 8.0) | 103.2 (10.1, 224.2) | 9.8 (1.0, 20.4) | 157.5 (15.8, 325.0) | 1.41 (1.38, 1.45) |
| Equatorial Guinea | 0.1 (0.0, 0.3) | 51.1 (5.1, 106.7) | 1.0 (0.1, 2.1) | 110.9 (10.7, 230.0) | 2.46 (2.39, 2.54) |
| Eritrea | 0.2 (0.0, 0.5) | 13.6 (1.5, 27.5) | 1.1 (0.1, 2.3) | 26.6 (2.6, 56.1) | 2.13 (2.08, 2.18) |
| Estonia | 3.2 (0.3, 6.7) | 164.0 (15.9, 344.6) | 4.1 (0.4, 8.5) | 200.4 (18.9, 420.2) | 0.75 (0.71, 0.79) |
| Eswatini | 0.3 (0.0, 0.7) | 86.3 (8.7, 177.3) | 1.0 (0.1, 1.9) | 132.5 (14.0, 259.4) | 1.37 (1.34, 1.39) |
| Ethiopia | 6.6 (0.8, 13.2) | 24.0 (2.9, 48.0) | 20.0 (2.2, 40.1) | 31.9 (3.5, 63.2) | 0.86 (0.83, 0.89) |
| Fiji | 0.6 (0.1, 1.2) | 109.1 (10.7, 228.9) | 1.4 (0.1, 2.8) | 153.7 (16.0, 309.2) | 1.11 (1.09, 1.13) |
| Finland | 6.2 (0.6, 13.0) | 95.4 (9.6, 199.7) | 10.8 (1.1, 22.5) | 127.6 (12.2, 270.3) | 0.98 (0.90, 1.05) |
| France | 52.8 (5.6, 106.1) | 73.5 (7.8, 147.7) | 126.3 (12.1, 267.1) | 131.3 (12.3, 282.1) | 1.92 (1.84, 2.01) |
| Gabon | 0.5 (0.0, 1.0) | 73.9 (7.4, 156.0) | 1.8 (0.2, 3.7) | 129.0 (13.2, 268.6) | 1.81 (1.80, 1.82) |
| Gambia | 0.3 (0.0, 0.5) | 53.1 (5.4, 107.0) | 1.1 (0.1, 2.3) | 77.5 (7.6, 165.0) | 1.20 (1.15, 1.24) |
| Georgia | 7.4 (0.7, 15.6) | 121.1 (12.0, 253.5) | 6.9 (0.7, 14.4) | 139.9 (13.6, 292.5) | 0.40 (0.34, 0.45) |
| Germany | 154.6 (14.9, 328.7) | 141.7 (13.6, 297.2) | 221.9 (21.4, 451.4) | 168.1 (15.8, 351.0) | 0.61 (0.57, 0.65) |
| Ghana | 3.2 (0.3, 6.7) | 36.5 (3.9, 74.8) | 18.3 (1.8, 39.3) | 77.3 (7.8, 163.7) | 2.41 (2.32, 2.50) |
| Greece | 12.5 (1.3, 26.5) | 93.5 (9.6, 198.3) | 23.3 (2.4, 48.1) | 145.9 (14.6, 308.3) | 1.39 (1.33, 1.45) |
| Greenland | 0.1 (0.0, 0.2) | 136.4 (12.8, 293.3) | 0.1 (0.0, 0.2) | 153.1 (15.0, 316.8) | 0.46 (0.41, 0.52) |
| Grenada | 0.0 (0.0, 0.1) | 65.6 (6.5, 140.3) | 0.1 (0.0, 0.3) | 104.9 (10.4, 215.1) | 1.57 (1.54, 1.61) |
| Guam | 0.1 (0.0, 0.3) | 108.2 (10.5, 230.3) | 0.3 (0.0, 0.5) | 135.1 (13.3, 274.4) | 0.72 (0.70, 0.73) |
| Guatemala | 5.6 (0.6, 11.9) | 116.2 (11.7, 244.0) | 21.5 (2.2, 45.2) | 161.3 (16.2, 339.1) | 1.18 (1.13, 1.23) |
| Guinea | 1.6 (0.2, 3.4) | 42.4 (4.3, 88.2) | 4.9 (0.5, 10.2) | 63.7 (6.2, 135.0) | 1.25 (1.21, 1.29) |
| Guinea-Bissau | 0.2 (0.0, 0.5) | 40.1 (4.2, 86.3) | 0.7 (0.1, 1.5) | 63.5 (6.4, 131.5) | 1.45 (1.43, 1.46) |
| Guyana | 0.3 (0.0, 0.7) | 60.0 (6.1, 128.1) | 0.7 (0.1, 1.4) | 92.4 (9.1, 195.1) | 1.42 (1.40, 1.44) |
| Haiti | 1.0 (0.1, 2.1) | 23.4 (2.6, 47.9) | 5.2 (0.5, 11.0) | 47.6 (4.7, 100.3) | 2.47 (2.41, 2.53) |
| Honduras | 1.9 (0.2, 3.9) | 71.3 (7.2, 150.4) | 9.2 (0.9, 19.3) | 114.0 (11.4, 243.0) | 1.54 (1.49, 1.59) |
| Hungary | 29.5 (2.9, 61.8) | 222.3 (21.6, 462.1) | 41.2 (4.1, 85.2) | 280.1 (27.4, 577.0) | 0.76 (0.76, 0.77) |
| Iceland | 0.4 (0.0, 0.8) | 133.1 (12.9, 287.9) | 0.7 (0.1, 1.5) | 161.5 (15.8, 337.9) | 0.61 (0.57, 0.65) |
| India | 113.1 (12.3, 225.5) | 18.3 (2.0, 36.6) | 554.4 (52.6, 1140.9) | 40.7 (3.9, 83.7) | 2.75 (2.63, 2.87) |
| Indonesia | 25.8 (2.8, 51.5) | 18.9 (2.1, 37.8) | 137.4 (13.3, 290.8) | 45.6 (4.3, 95.9) | 3.17 (3.00, 3.34) |
| Iran (Islamic Republic of) | 33.8 (3.3, 69.7) | 97.2 (9.5, 199.4) | 167.1 (16.5, 341.5) | 180.2 (17.8, 367.3) | 2.01 (1.96, 2.06) |
| Iraq | 14.7 (1.4, 32.5) | 136.6 (12.9, 296.6) | 55.2 (5.4, 114.2) | 163.5 (16.4, 333.6) | 0.61 (0.59, 0.63) |
| Ireland | 4.0 (0.4, 8.6) | 109.0 (10.8, 233.0) | 10.2 (1.0, 21.1) | 158.6 (15.3, 333.2) | 1.14 (1.04, 1.24) |
| Israel | 5.4 (0.5, 11.3) | 115.4 (11.3, 241.0) | 15.2 (1.5, 31.3) | 143.2 (14.4, 297.4) | 0.72 (0.69, 0.75) |
| Italy | 64.2 (6.4, 133.6) | 83.1 (8.2, 174.1) | 120.0 (11.8, 246.9) | 119.7 (11.7, 248.8) | 0.99 (0.92, 1.06) |
| Jamaica | 1.4 (0.1, 3.1) | 78.7 (7.7, 170.0) | 3.9 (0.4, 7.9) | 124.4 (12.5, 254.3) | 1.53 (1.46, 1.60) |
| Japan | 81.1 (9.4, 165.0) | 50.8 (5.9, 102.9) | 133.9 (14.8, 265.9) | 65.9 (7.1, 136.9) | 0.84 (0.78, 0.90) |
| Jordan | 2.7 (0.3, 5.7) | 138.4 (14.0, 287.3) | 21.6 (2.3, 42.5) | 207.1 (22.1, 402.9) | 1.27 (1.24, 1.31) |
| Kazakhstan | 17.1 (1.7, 35.3) | 125.7 (12.3, 262.0) | 32.0 (3.2, 65.6) | 165.2 (16.7, 337.7) | 0.85 (0.82, 0.87) |
| Kenya | 5.0 (0.5, 10.3) | 42.7 (4.4, 89.1) | 28.9 (2.8, 60.0) | 85.2 (8.5, 177.6) | 2.25 (2.24, 2.25) |
| Kiribati | 0.1 (0.0, 0.1) | 110.4 (11.2, 231.9) | 0.2 (0.0, 0.3) | 161.0 (16.6, 322.6) | 1.31 (1.25, 1.36) |
| Kuwait | 1.8 (0.2, 3.8) | 131.8 (13.1, 270.7) | 12.4 (1.3, 24.8) | 233.7 (25.8, 454.6) | 1.80 (1.78, 1.82) |
| Kyrgyzstan | 3.9 (0.4, 8.1) | 122.5 (12.5, 256.5) | 9.1 (0.9, 18.8) | 159.5 (15.6, 327.8) | 0.83 (0.79, 0.87) |
| Lao People’s Democratic Republic | 0.4 (0.1, 0.9) | 16.8 (2.0, 34.3) | 2.2 (0.2, 4.6) | 36.2 (3.6, 74.2) | 2.80 (2.67, 2.92) |
| Latvia | 5.4 (0.5, 11.3) | 161.3 (15.7, 340.3) | 5.9 (0.6, 12.0) | 192.0 (18.6, 391.0) | 0.54 (0.51, 0.57) |
| Lebanon | 2.6 (0.3, 5.5) | 107.6 (10.4, 224.3) | 10.4 (1.0, 21.0) | 171.2 (17.3, 344.5) | 1.56 (1.51, 1.61) |
| Lesotho | 0.8 (0.1, 1.6) | 81.7 (8.1, 168.5) | 1.4 (0.1, 3.0) | 108.1 (11.2, 223.1) | 0.83 (0.78, 0.88) |
| Liberia | 1.0 (0.1, 2.1) | 70.0 (7.0, 149.7) | 3.7 (0.4, 7.9) | 107.5 (10.8, 232.3) | 1.33 (1.31, 1.35) |
| Libya | 2.5 (0.2, 5.3) | 101.0 (10.2, 212.3) | 14.2 (1.5, 28.5) | 198.8 (21.2, 393.6) | 2.19 (2.15, 2.24) |
| Lithuania | 5.9 (0.6, 12.5) | 135.3 (13.6, 288.2) | 8.3 (0.8, 16.8) | 183.1 (18.0, 372.4) | 0.92 (0.88, 0.97) |
| Luxembourg | 0.5 (0.1, 1.1) | 103.6 (10.3, 219.0) | 1.3 (0.1, 2.7) | 143.6 (13.8, 301.4) | 1.03 (0.98, 1.07) |
| Madagascar | 1.4 (0.2, 3.1) | 22.9 (2.5, 49.2) | 6.9 (0.7, 14.5) | 44.0 (4.3, 92.8) | 2.04 (1.96, 2.11) |
| Malawi | 1.5 (0.2, 3.1) | 27.4 (3.2, 56.7) | 6.3 (0.6, 13.1) | 55.4 (5.6, 118.3) | 2.20 (2.11, 2.29) |
| Malaysia | 5.6 (0.6, 11.5) | 43.8 (4.4, 89.6) | 25.1 (2.5, 52.9) | 76.9 (7.6, 161.4) | 1.76 (1.67, 1.84) |
| Maldives | 0.0 (0.0, 0.1) | 28.4 (3.0, 59.0) | 0.3 (0.0, 0.7) | 58.5 (5.9, 125.7) | 2.54 (2.40, 2.67) |
| Mali | 1.4 (0.1, 2.9) | 28.7 (2.9, 58.4) | 5.6 (0.6, 11.8) | 45.2 (4.5, 96.7) | 1.45 (1.32, 1.58) |
| Malta | 0.4 (0.0, 0.7) | 86.3 (8.7, 176.9) | 1.0 (0.1, 2.2) | 149.8 (13.9, 316.3) | 1.71 (1.56, 1.86) |
| Marshall Islands | 0.0 (0.0, 0.1) | 118.6 (11.7, 249.8) | 0.1 (0.0, 0.1) | 146.7 (15.7, 293.7) | 0.67 (0.66, 0.68) |
| Mauritania | 0.9 (0.1, 1.9) | 72.8 (7.0, 151.4) | 3.0 (0.3, 6.3) | 107.7 (10.7, 227.7) | 1.19 (1.14, 1.23) |
| Mauritius | 0.4 (0.0, 0.9) | 48.4 (4.9, 101.8) | 1.4 (0.1, 2.9) | 82.5 (8.0, 171.4) | 1.79 (1.69, 1.89) |
| Mexico | 57.2 (5.6, 120.8) | 98.7 (9.9, 207.9) | 206.3 (21.0, 428.9) | 151.0 (15.4, 314.4) | 1.33 (1.21, 1.45) |
| Micronesia (Federated States of) | 0.1 (0.0, 0.2) | 125.3 (12.9, 259.4) | 0.2 (0.0, 0.3) | 168.2 (18.3, 332.9) | 0.97 (0.94, 1.01) |
| Monaco | 0.1 (0.0, 0.1) | 126.7 (12.1, 265.5) | 0.1 (0.0, 0.2) | 155.2 (14.9, 325.1) | 0.66 (0.64, 0.68) |
| Mongolia | 1.2 (0.1, 2.6) | 98.3 (9.9, 206.0) | 3.7 (0.4, 7.8) | 121.9 (11.9, 255.3) | 0.69 (0.66, 0.72) |
| Montenegro | 1.2 (0.1, 2.5) | 184.0 (17.7, 387.2) | 2.2 (0.2, 4.5) | 253.9 (25.7, 528.4) | 1.08 (1.05, 1.10) |
| Morocco | 15.6 (1.5, 33.3) | 88.2 (8.7, 187.6) | 56.1 (5.5, 115.0) | 147.1 (14.5, 302.3) | 1.76 (1.64, 1.87) |
| Mozambique | 2.3 (0.3, 4.7) | 29.6 (3.3, 60.8) | 10.5 (1.0, 21.9) | 63.9 (6.3, 133.4) | 2.55 (2.40, 2.69) |
| Myanmar | 5.3 (0.6, 11.1) | 18.3 (1.9, 37.6) | 17.2 (1.8, 35.8) | 30.8 (3.3, 63.6) | 1.66 (1.60, 1.72) |
| Namibia | 0.4 (0.0, 0.9) | 54.9 (5.1, 111.4) | 1.9 (0.2, 3.8) | 106.0 (10.1, 217.8) | 2.21 (2.14, 2.27) |
| Nauru | 0.0 (0.0, 0.0) | 151.4 (15.9, 303.0) | 0.0 (0.0, 0.0) | 196.3 (22.1, 386.8) | 0.86 (0.80, 0.91) |
| Nepal | 4.1 (0.4, 8.2) | 30.7 (3.3, 61.6) | 17.5 (1.7, 36.8) | 62.6 (6.3, 129.9) | 2.64 (2.51, 2.78) |
| Netherlands | 14.6 (1.4, 29.9) | 80.0 (7.9, 162.9) | 29.5 (2.9, 62.6) | 116.9 (11.1, 251.4) | 1.31 (1.28, 1.35) |
| New Zealand | 6.2 (0.6, 12.6) | 167.4 (16.1, 342.1) | 14.3 (1.4, 29.3) | 216.5 (20.5, 453.0) | 0.96 (0.92, 1.00) |
| Nicaragua | 2.3 (0.2, 5.0) | 108.5 (10.4, 232.7) | 9.2 (0.9, 18.7) | 152.7 (15.7, 309.4) | 1.14 (1.13, 1.15) |
| Niger | 1.5 (0.2, 3.0) | 36.4 (3.8, 76.9) | 5.6 (0.6, 11.9) | 48.9 (4.9, 103.5) | 0.99 (0.97, 1.02) |
| Nigeria | 22.6 (2.4, 46.0) | 41.7 (4.4, 83.8) | 105.1 (10.0, 223.8) | 82.0 (8.1, 171.8) | 2.15 (2.10, 2.20) |
| Niue | 0.0 (0.0, 0.0) | 121.8 (12.3, 257.2) | 0.0 (0.0, 0.0) | 164.8 (17.7, 327.7) | 0.99 (0.97, 1.02) |
| North Macedonia | 3.2 (0.3, 6.7) | 156.0 (14.9, 330.3) | 6.3 (0.6, 13.0) | 203.5 (20.0, 421.7) | 0.89 (0.87, 0.91) |
| Northern Mariana Islands | 0.1 (0.0, 0.1) | 133.4 (13.4, 275.0) | 0.1 (0.0, 0.2) | 175.7 (18.9, 342.8) | 0.88 (0.82, 0.93) |
| Norway | 5.0 (0.5, 10.1) | 92.7 (9.4, 190.9) | 8.7 (0.8, 18.2) | 115.3 (11.0, 245.3) | 0.62 (0.59, 0.65) |
| Oman | 1.1 (0.1, 2.3) | 86.2 (8.3, 178.0) | 8.2 (0.8, 16.5) | 184.3 (19.2, 367.3) | 2.61 (2.57, 2.64) |
| Pakistan | 22.1 (2.5, 44.5) | 31.6 (3.6, 64.0) | 134.5 (13.0, 285.5) | 77.9 (7.7, 163.9) | 3.54 (3.34, 3.75) |
| Palau | 0.0 (0.0, 0.0) | 125.8 (12.5, 255.1) | 0.0 (0.0, 0.1) | 157.5 (16.2, 314.9) | 0.73 (0.71, 0.76) |
| Palestine | 1.5 (0.1, 3.1) | 133.1 (13.7, 277.6) | 7.2 (0.7, 14.4) | 192.9 (20.0, 378.8) | 1.21 (1.20, 1.23) |
| Panama | 1.3 (0.1, 2.8) | 77.0 (8.3, 165.8) | 6.1 (0.6, 12.8) | 139.6 (14.0, 290.2) | 2.00 (1.93, 2.07) |
| Papua New Guinea | 1.1 (0.1, 2.2) | 40.8 (4.4, 83.2) | 4.9 (0.5, 10.6) | 61.9 (6.3, 132.9) | 1.38 (1.31, 1.45) |
| Paraguay | 2.1 (0.2, 4.5) | 79.7 (7.7, 168.4) | 8.5 (0.8, 17.6) | 127.3 (12.1, 259.5) | 1.55 (1.44, 1.66) |
| Peru | 8.6 (0.8, 18.0) | 57.6 (5.6, 121.0) | 34.7 (3.5, 72.9) | 95.4 (9.6, 200.0) | 1.71 (1.67, 1.75) |
| Philippines | 12.5 (1.3, 26.6) | 30.5 (3.2, 63.7) | 55.8 (5.1, 119.0) | 55.6 (5.2, 117.9) | 1.87 (1.80, 1.95) |
| Poland | 66.6 (6.5, 142.9) | 155.1 (15.1, 329.5) | 113.8 (11.4, 233.4) | 194.2 (19.1, 399.4) | 0.69 (0.64, 0.74) |
| Portugal | 12.0 (1.2, 25.4) | 96.6 (9.8, 202.8) | 25.4 (2.4, 52.0) | 151.2 (14.1, 318.4) | 1.35 (1.25, 1.46) |
| Puerto Rico | 3.7 (0.4, 7.9) | 101.6 (10.1, 219.3) | 6.6 (0.7, 13.1) | 138.1 (14.0, 277.5) | 0.95 (0.89, 1.01) |
| Qatar | 0.5 (0.0, 1.0) | 125.9 (12.5, 261.9) | 6.8 (0.7, 13.9) | 211.6 (22.8, 422.2) | 1.61 (1.59, 1.64) |
| Republic of Korea | 14.2 (1.7, 29.3) | 35.0 (4.4, 71.2) | 48.6 (5.1, 100.3) | 60.7 (6.3, 124.1) | 1.83 (1.78, 1.88) |
| Republic of Moldova | 6.8 (0.7, 14.4) | 149.6 (14.9, 314.5) | 11.4 (1.1, 22.7) | 211.3 (21.0, 419.4) | 1.05 (0.98, 1.12) |
| Romania | 47.0 (4.5, 102.0) | 173.6 (16.7, 373.4) | 65.0 (6.4, 134.8) | 229.0 (22.3, 481.1) | 0.82 (0.76, 0.89) |
| Russian Federation | 269.5 (26.4, 559.4) | 151.6 (14.8, 316.0) | 434.8 (44.6, 897.7) | 204.3 (20.5, 421.3) | 1.10 (1.06, 1.14) |
| Rwanda | 1.1 (0.1, 2.1) | 27.2 (3.2, 54.2) | 4.4 (0.4, 9.0) | 48.6 (4.8, 98.2) | 1.77 (1.67, 1.87) |
| Saint Kitts and Nevis | 0.0 (0.0, 0.1) | 71.4 (7.3, 152.4) | 0.1 (0.0, 0.2) | 113.7 (11.6, 233.2) | 1.52 (1.49, 1.55) |
| Saint Lucia | 0.1 (0.0, 0.1) | 69.9 (6.8, 151.0) | 0.2 (0.0, 0.5) | 106.4 (10.6, 221.9) | 1.40 (1.38, 1.43) |
| Saint Vincent and the Grenadines | 0.0 (0.0, 0.1) | 46.2 (5.0, 97.7) | 0.1 (0.0, 0.2) | 81.6 (7.9, 172.2) | 1.88 (1.87, 1.89) |
| Samoa | 0.2 (0.0, 0.3) | 154.4 (16.1, 310.0) | 0.3 (0.0, 0.6) | 183.0 (20.7, 351.5) | 0.49 (0.44, 0.54) |
| San Marino | 0.0 (0.0, 0.1) | 115.7 (10.9, 244.9) | 0.1 (0.0, 0.2) | 150.4 (14.4, 313.2) | 0.85 (0.83, 0.88) |
| Sao Tome and Principe | 0.0 (0.0, 0.1) | 56.6 (5.7, 119.6) | 0.1 (0.0, 0.3) | 87.7 (9.1, 185.8) | 1.38 (1.35, 1.41) |
| Saudi Arabia | 11.5 (1.1, 24.8) | 114.4 (11.2, 239.4) | 80.8 (8.8, 163.2) | 207.8 (22.4, 413.6) | 1.93 (1.90, 1.95) |
| Senegal | 2.1 (0.2, 4.4) | 50.4 (5.1, 106.7) | 7.1 (0.7, 15.2) | 69.2 (6.6, 146.8) | 0.92 (0.88, 0.97) |
| Serbia | 17.6 (1.8, 38.4) | 151.7 (15.0, 330.2) | 31.4 (3.2, 64.9) | 237.7 (23.6, 493.3) | 1.46 (1.41, 1.50) |
| Seychelles | 0.0 (0.0, 0.1) | 65.2 (6.5, 139.4) | 0.1 (0.0, 0.3) | 104.5 (10.6, 216.2) | 1.48 (1.41, 1.55) |
| Sierra Leone | 0.9 (0.1, 1.9) | 38.2 (4.1, 77.8) | 3.2 (0.3, 6.7) | 59.6 (6.0, 128.0) | 1.34 (1.24, 1.44) |
| Singapore | 1.0 (0.1, 2.1) | 34.0 (4.0, 69.2) | 7.1 (0.7, 15.2) | 87.3 (8.2, 186.1) | 3.19 (3.11, 3.27) |
| Slovakia | 11.5 (1.1, 24.6) | 197.8 (18.6, 418.3) | 18.6 (1.9, 38.8) | 226.5 (22.6, 476.5) | 0.46 (0.44, 0.48) |
| Slovenia | 4.1 (0.4, 8.6) | 170.4 (16.4, 362.3) | 7.0 (0.7, 14.5) | 212.6 (20.9, 444.2) | 0.72 (0.70, 0.73) |
| Solomon Islands | 0.1 (0.0, 0.3) | 67.3 (6.6, 148.2) | 0.5 (0.1, 1.1) | 103.5 (10.3, 216.8) | 1.47 (1.45, 1.49) |
| Somalia | 1.4 (0.1, 2.8) | 32.4 (3.5, 65.8) | 6.3 (0.6, 13.2) | 55.5 (5.5, 117.5) | 1.73 (1.68, 1.79) |
| South Africa | 25.5 (2.5, 52.9) | 103.8 (10.5, 213.2) | 73.5 (7.6, 147.9) | 136.9 (14.3, 272.5) | 0.91 (0.90, 0.92) |
| South Sudan | 0.6 (0.1, 1.2) | 18.3 (2.3, 37.6) | 1.5 (0.2, 2.9) | 25.5 (2.9, 51.0) | 1.02 (0.96, 1.08) |
| Spain | 45.6 (4.4, 96.9) | 95.1 (9.3, 203.3) | 91.9 (9.2, 192.4) | 128.5 (12.6, 269.8) | 1.14 (1.07, 1.20) |
| Sri Lanka | 3.8 (0.4, 8.0) | 27.7 (2.7, 57.6) | 14.0 (1.3, 30.1) | 53.7 (4.9, 114.4) | 2.10 (2.01, 2.18) |
| Sudan | 10.6 (1.0, 22.2) | 86.6 (8.4, 179.5) | 47.5 (4.8, 99.1) | 157.5 (15.8, 326.1) | 1.96 (1.93, 1.99) |
| Suriname | 0.1 (0.0, 0.3) | 44.0 (4.5, 91.3) | 0.5 (0.1, 1.1) | 80.9 (7.8, 176.3) | 1.99 (1.97, 2.02) |
| Sweden | 7.2 (0.7, 14.9) | 58.7 (5.6, 122.2) | 15.4 (1.5, 31.8) | 102.5 (10.1, 213.9) | 1.86 (1.69, 2.04) |
| Switzerland | 9.0 (0.9, 18.2) | 100.7 (9.6, 203.5) | 16.0 (1.6, 33.5) | 119.2 (11.5, 250.1) | 0.75 (0.64, 0.86) |
| Syrian Arab Republic | 7.7 (0.8, 16.6) | 111.8 (11.2, 240.9) | 29.6 (3.2, 58.2) | 209.7 (22.6, 421.9) | 2.06 (2.04, 2.07) |
| Taiwan (Province of China) | 6.6 (0.7, 13.3) | 36.0 (3.9, 71.9) | 36.6 (3.3, 75.6) | 102.0 (9.1, 210.1) | 3.59 (3.46, 3.73) |
| Tajikistan | 3.4 (0.3, 7.2) | 109.9 (10.8, 231.4) | 10.1 (1.0, 21.4) | 133.1 (13.3, 278.6) | 0.60 (0.56, 0.63) |
| Thailand | 10.9 (1.1, 23.0) | 23.0 (2.4, 47.8) | 59.7 (5.9, 126.3) | 61.9 (6.1, 132.8) | 3.40 (3.33, 3.46) |
| Timor-Leste | 0.0 (0.0, 0.1) | 9.2 (1.2, 18.9) | 0.2 (0.0, 0.4) | 17.8 (2.0, 35.4) | 2.21 (2.09, 2.32) |
| Togo | 0.8 (0.1, 1.7) | 45.7 (4.6, 96.0) | 4.2 (0.4, 8.8) | 75.2 (7.2, 158.2) | 1.58 (1.55, 1.60) |
| Tokelau | 0.0 (0.0, 0.0) | 115.8 (11.6, 246.6) | 0.0 (0.0, 0.0) | 160.1 (17.2, 325.8) | 1.07 (1.03, 1.10) |
| Tonga | 0.1 (0.0, 0.2) | 165.3 (18.1, 327.5) | 0.2 (0.0, 0.3) | 207.5 (23.9, 396.7) | 0.72 (0.70, 0.75) |
| Trinidad and Tobago | 0.8 (0.1, 1.7) | 81.7 (7.9, 172.3) | 2.1 (0.2, 4.3) | 118.4 (11.7, 243.9) | 1.19 (1.18, 1.20) |
| Tunisia | 4.9 (0.5, 10.0) | 80.7 (8.0, 167.1) | 21.3 (2.1, 43.5) | 156.8 (15.5, 319.1) | 2.18 (2.17, 2.20) |
| Turkmenistan | 2.5 (0.3, 5.2) | 110.4 (11.3, 233.3) | 6.0 (0.6, 12.7) | 128.2 (12.7, 265.4) | 0.48 (0.46, 0.50) |
| Tuvalu | 0.0 (0.0, 0.0) | 115.1 (11.2, 245.8) | 0.0 (0.0, 0.0) | 160.7 (17.1, 319.6) | 1.16 (1.13, 1.18) |
| Türkiye | 49.3 (5.0, 102.7) | 116.5 (11.9, 239.2) | 177.3 (18.5, 357.4) | 184.7 (19.1, 374.5) | 1.51 (1.42, 1.61) |
| Uganda | 2.7 (0.3, 5.4) | 30.4 (3.5, 61.3) | 13.1 (1.3, 26.8) | 57.3 (6.0, 116.1) | 2.15 (2.12, 2.19) |
| Ukraine | 120.6 (11.7, 248.3) | 179.3 (17.3, 367.9) | 152.0 (15.3, 311.3) | 226.1 (22.3, 463.8) | 0.82 (0.75, 0.89) |
| United Arab Emirates | 1.4 (0.1, 3.0) | 93.2 (9.1, 197.0) | 24.3 (2.6, 48.5) | 193.5 (20.1, 379.1) | 2.37 (2.34, 2.39) |
| United Kingdom | 84.8 (8.4, 178.7) | 118.0 (11.5, 249.2) | 157.2 (15.8, 320.6) | 164.5 (16.2, 341.0) | 1.25 (1.16, 1.34) |
| United Republic of Tanzania | 6.0 (0.6, 12.1) | 43.7 (4.4, 88.9) | 31.5 (2.9, 66.4) | 89.6 (8.6, 189.8) | 2.49 (2.40, 2.57) |
| United States Virgin Islands | 0.1 (0.0, 0.2) | 111.0 (10.9, 233.8) | 0.2 (0.0, 0.3) | 135.9 (14.0, 270.6) | 0.66 (0.65, 0.68) |
| United States of America | 529.6 (49.9, 1129.8) | 184.8 (17.5, 395.8) | 1048.2 (105.4, 2097.5) | 241.0 (24.3, 486.0) | 0.97 (0.88, 1.06) |
| Uruguay | 3.9 (0.4, 8.4) | 112.2 (11.1, 238.8) | 7.4 (0.8, 15.3) | 172.0 (17.1, 359.9) | 1.38 (1.33, 1.43) |
| Uzbekistan | 14.5 (1.4, 30.4) | 113.8 (11.1, 237.8) | 47.2 (4.6, 99.3) | 149.4 (14.6, 313.1) | 0.99 (0.95, 1.03) |
| Vanuatu | 0.1 (0.0, 0.1) | 67.8 (6.5, 145.3) | 0.3 (0.0, 0.5) | 106.2 (10.5, 219.8) | 1.45 (1.37, 1.52) |
| Venezuela (Bolivarian Republic of) | 13.2 (1.2, 28.8) | 100.0 (9.5, 215.0) | 42.0 (4.3, 84.2) | 140.5 (14.2, 284.6) | 1.09 (1.08, 1.11) |
| Viet Nam | 4.1 (0.5, 8.1) | 9.0 (1.2, 17.7) | 24.3 (2.6, 50.7) | 21.8 (2.4, 45.3) | 3.37 (3.14, 3.60) |
| Yemen | 3.5 (0.4, 7.4) | 50.9 (5.4, 106.6) | 22.3 (2.0, 46.0) | 101.9 (9.1, 208.7) | 2.30 (2.17, 2.44) |
| Zambia | 1.1 (0.1, 2.2) | 26.6 (3.0, 55.4) | 8.1 (0.8, 17.2) | 74.5 (7.0, 156.5) | 3.23 (3.15, 3.30) |
| Zimbabwe | 2.4 (0.2, 5.1) | 46.4 (4.6, 97.3) | 9.6 (1.0, 20.3) | 102.3 (10.2, 216.0) | 2.61 (2.51, 2.72) |

***Footnote***: YLDs: Years lived with disability; ASYR: Age-standardized YLD rates; EAPC: Estimated annual percentage change.

## Supplementary Table S2. Estimated Annual Percentage Change in Age-Standardized Years Lived with Disability Rates for Low Back Pain Attributable to High Body Mass Index by Five-Year Age Groups in Global and Five Socio-Demographic Index Regions for Both Males and Females, 1990–2021.

| Region | age_name | eval | elower | eupper | Sex |
| --- | --- | --- | --- | --- | --- |
| Global | 20-24 years | 2.2 | 2.2 | 2.3 | Both |
| Global | 25-29 years | 2.0 | 1.9 | 2.1 | Both |
| Global | 30-34 years | 1.6 | 1.5 | 1.7 | Both |
| Global | 35-39 years | 1.4 | 1.3 | 1.5 | Both |
| Global | 40-44 years | 1.2 | 1.1 | 1.3 | Both |
| Global | 45-49 years | 1.1 | 1.0 | 1.1 | Both |
| Global | 50-54 years | 1.0 | 1.0 | 1.1 | Both |
| Global | 55-59 years | 1.1 | 1.1 | 1.2 | Both |
| Global | 60-64 years | 1.1 | 1.0 | 1.1 | Both |
| Global | 65-69 years | 0.9 | 0.8 | 0.9 | Both |
| Global | 70-74 years | 0.8 | 0.8 | 0.9 | Both |
| Global | 75-79 years | 0.7 | 0.6 | 0.8 | Both |
| Global | 80-84 years | 0.7 | 0.6 | 0.8 | Both |
| Global | 85-89 years | 0.7 | 0.6 | 0.8 | Both |
| Global | 90-94 years | 0.8 | 0.7 | 0.8 | Both |
| Global | 95+ years | 0.7 | 0.7 | 0.7 | Both |
| High SDI | 20-24 years | 1.9 | 1.8 | 2.0 | Both |
| High SDI | 25-29 years | 1.5 | 1.4 | 1.5 | Both |
| High SDI | 30-34 years | 1.3 | 1.3 | 1.3 | Both |
| High SDI | 35-39 years | 1.2 | 1.1 | 1.2 | Both |
| High SDI | 40-44 years | 1.0 | 1.0 | 1.1 | Both |
| High SDI | 45-49 years | 0.9 | 0.8 | 1.0 | Both |
| High SDI | 50-54 years | 0.9 | 0.8 | 0.9 | Both |
| High SDI | 55-59 years | 0.9 | 0.9 | 1.0 | Both |
| High SDI | 60-64 years | 1.0 | 0.9 | 1.0 | Both |
| High SDI | 65-69 years | 1.0 | 1.0 | 1.1 | Both |
| High SDI | 70-74 years | 1.0 | 0.9 | 1.0 | Both |
| High SDI | 75-79 years | 0.9 | 0.8 | 0.9 | Both |
| High SDI | 80-84 years | 0.7 | 0.7 | 0.8 | Both |
| High SDI | 85-89 years | 0.7 | 0.6 | 0.7 | Both |
| High SDI | 90-94 years | 0.6 | 0.6 | 0.7 | Both |
| High SDI | 95+ years | 0.5 | 0.4 | 0.5 | Both |
| High-middle SDI | 20-24 years | 2.9 | 2.7 | 3.1 | Both |
| High-middle SDI | 25-29 years | 2.4 | 2.2 | 2.6 | Both |
| High-middle SDI | 30-34 years | 1.7 | 1.5 | 1.9 | Both |
| High-middle SDI | 35-39 years | 1.4 | 1.1 | 1.6 | Both |
| High-middle SDI | 40-44 years | 1.0 | 0.7 | 1.2 | Both |
| High-middle SDI | 45-49 years | 0.7 | 0.6 | 0.8 | Both |
| High-middle SDI | 50-54 years | 0.5 | 0.3 | 0.6 | Both |
| High-middle SDI | 55-59 years | 0.5 | 0.4 | 0.7 | Both |
| High-middle SDI | 60-64 years | 0.5 | 0.3 | 0.6 | Both |
| High-middle SDI | 65-69 years | 0.3 | 0.2 | 0.4 | Both |
| High-middle SDI | 70-74 years | 0.4 | 0.3 | 0.6 | Both |
| High-middle SDI | 75-79 years | 0.5 | 0.3 | 0.6 | Both |
| High-middle SDI | 80-84 years | 0.5 | 0.4 | 0.6 | Both |
| High-middle SDI | 85-89 years | 0.5 | 0.4 | 0.6 | Both |
| High-middle SDI | 90-94 years | 0.5 | 0.4 | 0.6 | Both |
| High-middle SDI | 95+ years | 0.6 | 0.5 | 0.6 | Both |
| Low SDI | 20-24 years | 1.8 | 1.7 | 1.9 | Both |
| Low SDI | 25-29 years | 2.2 | 2.1 | 2.3 | Both |
| Low SDI | 30-34 years | 2.3 | 2.3 | 2.4 | Both |
| Low SDI | 35-39 years | 2.3 | 2.2 | 2.4 | Both |
| Low SDI | 40-44 years | 2.3 | 2.2 | 2.4 | Both |
| Low SDI | 45-49 years | 2.0 | 1.9 | 2.1 | Both |
| Low SDI | 50-54 years | 1.9 | 1.8 | 2.0 | Both |
| Low SDI | 55-59 years | 1.9 | 1.9 | 1.9 | Both |
| Low SDI | 60-64 years | 1.9 | 1.8 | 1.9 | Both |
| Low SDI | 65-69 years | 1.7 | 1.7 | 1.8 | Both |
| Low SDI | 70-74 years | 1.8 | 1.7 | 1.8 | Both |
| Low SDI | 75-79 years | 1.7 | 1.7 | 1.8 | Both |
| Low SDI | 80-84 years | 1.7 | 1.6 | 1.8 | Both |
| Low SDI | 85-89 years | 1.9 | 1.7 | 2.0 | Both |
| Low SDI | 90-94 years | 1.8 | 1.6 | 2.1 | Both |
| Low SDI | 95+ years | 1.7 | 1.4 | 2.1 | Both |
| Low-middle SDI | 20-24 years | 2.7 | 2.7 | 2.8 | Both |
| Low-middle SDI | 25-29 years | 2.7 | 2.7 | 2.8 | Both |
| Low-middle SDI | 30-34 years | 2.6 | 2.5 | 2.7 | Both |
| Low-middle SDI | 35-39 years | 2.5 | 2.4 | 2.6 | Both |
| Low-middle SDI | 40-44 years | 2.6 | 2.5 | 2.6 | Both |
| Low-middle SDI | 45-49 years | 2.3 | 2.2 | 2.3 | Both |
| Low-middle SDI | 50-54 years | 2.5 | 2.4 | 2.6 | Both |
| Low-middle SDI | 55-59 years | 2.7 | 2.6 | 2.8 | Both |
| Low-middle SDI | 60-64 years | 2.7 | 2.6 | 2.8 | Both |
| Low-middle SDI | 65-69 years | 2.4 | 2.3 | 2.5 | Both |
| Low-middle SDI | 70-74 years | 2.0 | 2.0 | 2.1 | Both |
| Low-middle SDI | 75-79 years | 1.9 | 1.8 | 1.9 | Both |
| Low-middle SDI | 80-84 years | 1.9 | 1.8 | 1.9 | Both |
| Low-middle SDI | 85-89 years | 2.0 | 1.9 | 2.1 | Both |
| Low-middle SDI | 90-94 years | 2.2 | 2.1 | 2.2 | Both |
| Low-middle SDI | 95+ years | 2.8 | 2.6 | 2.9 | Both |
| Middle SDI | 20-24 years | 3.2 | 3.1 | 3.3 | Both |
| Middle SDI | 25-29 years | 2.9 | 2.8 | 3.1 | Both |
| Middle SDI | 30-34 years | 2.4 | 2.3 | 2.6 | Both |
| Middle SDI | 35-39 years | 2.2 | 2.1 | 2.4 | Both |
| Middle SDI | 40-44 years | 2.1 | 2.1 | 2.2 | Both |
| Middle SDI | 45-49 years | 2.0 | 1.9 | 2.0 | Both |
| Middle SDI | 50-54 years | 2.0 | 2.0 | 2.1 | Both |
| Middle SDI | 55-59 years | 2.1 | 2.0 | 2.1 | Both |
| Middle SDI | 60-64 years | 2.1 | 2.0 | 2.2 | Both |
| Middle SDI | 65-69 years | 2.1 | 2.0 | 2.1 | Both |
| Middle SDI | 70-74 years | 2.0 | 2.0 | 2.1 | Both |
| Middle SDI | 75-79 years | 2.0 | 1.9 | 2.0 | Both |
| Middle SDI | 80-84 years | 2.1 | 2.0 | 2.1 | Both |
| Middle SDI | 85-89 years | 1.9 | 1.9 | 2.0 | Both |
| Middle SDI | 90-94 years | 1.8 | 1.8 | 1.9 | Both |
| Middle SDI | 95+ years | 1.8 | 1.8 | 1.9 | Both |
| Global | 20-24 years | 2.2 | 2.2 | 2.2 | Male |
| Global | 25-29 years | 1.9 | 1.8 | 2.0 | Male |
| Global | 30-34 years | 1.5 | 1.4 | 1.6 | Male |
| Global | 35-39 years | 1.4 | 1.3 | 1.5 | Male |
| Global | 40-44 years | 1.2 | 1.2 | 1.3 | Male |
| Global | 45-49 years | 1.2 | 1.1 | 1.2 | Male |
| Global | 50-54 years | 1.1 | 1.0 | 1.2 | Male |
| Global | 55-59 years | 1.2 | 1.1 | 1.3 | Male |
| Global | 60-64 years | 1.2 | 1.1 | 1.3 | Male |
| Global | 65-69 years | 1.1 | 1.1 | 1.1 | Male |
| Global | 70-74 years | 1.2 | 1.2 | 1.3 | Male |
| Global | 75-79 years | 1.2 | 1.1 | 1.3 | Male |
| Global | 80-84 years | 1.3 | 1.2 | 1.3 | Male |
| Global | 85-89 years | 1.4 | 1.4 | 1.5 | Male |
| Global | 90-94 years | 1.6 | 1.6 | 1.7 | Male |
| Global | 95+ years | 1.6 | 1.6 | 1.7 | Male |
| High SDI | 20-24 years | 1.9 | 1.8 | 1.9 | Male |
| High SDI | 25-29 years | 1.4 | 1.3 | 1.4 | Male |
| High SDI | 30-34 years | 1.2 | 1.2 | 1.3 | Male |
| High SDI | 35-39 years | 1.2 | 1.1 | 1.2 | Male |
| High SDI | 40-44 years | 1.1 | 1.0 | 1.1 | Male |
| High SDI | 45-49 years | 1.0 | 0.9 | 1.1 | Male |
| High SDI | 50-54 years | 1.0 | 0.9 | 1.0 | Male |
| High SDI | 55-59 years | 1.1 | 1.0 | 1.1 | Male |
| High SDI | 60-64 years | 1.2 | 1.1 | 1.3 | Male |
| High SDI | 65-69 years | 1.3 | 1.2 | 1.4 | Male |
| High SDI | 70-74 years | 1.4 | 1.3 | 1.5 | Male |
| High SDI | 75-79 years | 1.3 | 1.3 | 1.4 | Male |
| High SDI | 80-84 years | 1.2 | 1.2 | 1.3 | Male |
| High SDI | 85-89 years | 1.2 | 1.2 | 1.3 | Male |
| High SDI | 90-94 years | 1.2 | 1.2 | 1.3 | Male |
| High SDI | 95+ years | 1.0 | 0.9 | 1.1 | Male |
| High-middle SDI | 20-24 years | 2.6 | 2.4 | 2.8 | Male |
| High-middle SDI | 25-29 years | 2.3 | 2.1 | 2.4 | Male |
| High-middle SDI | 30-34 years | 1.8 | 1.7 | 2.0 | Male |
| High-middle SDI | 35-39 years | 1.7 | 1.5 | 1.8 | Male |
| High-middle SDI | 40-44 years | 1.4 | 1.3 | 1.6 | Male |
| High-middle SDI | 45-49 years | 1.1 | 1.1 | 1.2 | Male |
| High-middle SDI | 50-54 years | 0.7 | 0.6 | 0.8 | Male |
| High-middle SDI | 55-59 years | 0.7 | 0.6 | 0.8 | Male |
| High-middle SDI | 60-64 years | 0.6 | 0.5 | 0.7 | Male |
| High-middle SDI | 65-69 years | 0.5 | 0.4 | 0.6 | Male |
| High-middle SDI | 70-74 years | 0.7 | 0.6 | 0.9 | Male |
| High-middle SDI | 75-79 years | 0.8 | 0.7 | 1.0 | Male |
| High-middle SDI | 80-84 years | 0.8 | 0.7 | 1.0 | Male |
| High-middle SDI | 85-89 years | 0.9 | 0.8 | 1.0 | Male |
| High-middle SDI | 90-94 years | 1.1 | 1.1 | 1.2 | Male |
| High-middle SDI | 95+ years | 1.4 | 1.3 | 1.5 | Male |
| Low SDI | 20-24 years | 2.1 | 2.1 | 2.2 | Male |
| Low SDI | 25-29 years | 2.6 | 2.6 | 2.7 | Male |
| Low SDI | 30-34 years | 2.7 | 2.7 | 2.8 | Male |
| Low SDI | 35-39 years | 2.8 | 2.7 | 2.9 | Male |
| Low SDI | 40-44 years | 2.8 | 2.7 | 2.9 | Male |
| Low SDI | 45-49 years | 2.7 | 2.6 | 2.8 | Male |
| Low SDI | 50-54 years | 2.6 | 2.5 | 2.6 | Male |
| Low SDI | 55-59 years | 2.5 | 2.4 | 2.5 | Male |
| Low SDI | 60-64 years | 2.4 | 2.3 | 2.4 | Male |
| Low SDI | 65-69 years | 2.2 | 2.2 | 2.3 | Male |
| Low SDI | 70-74 years | 2.2 | 2.1 | 2.2 | Male |
| Low SDI | 75-79 years | 2.2 | 2.2 | 2.3 | Male |
| Low SDI | 80-84 years | 2.2 | 2.1 | 2.3 | Male |
| Low SDI | 85-89 years | 2.4 | 2.3 | 2.6 | Male |
| Low SDI | 90-94 years | 2.4 | 2.1 | 2.8 | Male |
| Low SDI | 95+ years | 2.5 | 2.0 | 2.9 | Male |
| Low-middle SDI | 20-24 years | 3.1 | 3.1 | 3.2 | Male |
| Low-middle SDI | 25-29 years | 3.1 | 3.0 | 3.2 | Male |
| Low-middle SDI | 30-34 years | 2.9 | 2.8 | 3.0 | Male |
| Low-middle SDI | 35-39 years | 2.9 | 2.8 | 3.0 | Male |
| Low-middle SDI | 40-44 years | 2.7 | 2.7 | 2.8 | Male |
| Low-middle SDI | 45-49 years | 2.7 | 2.6 | 2.7 | Male |
| Low-middle SDI | 50-54 years | 2.7 | 2.6 | 2.8 | Male |
| Low-middle SDI | 55-59 years | 3.0 | 2.9 | 3.1 | Male |
| Low-middle SDI | 60-64 years | 2.9 | 2.9 | 3.0 | Male |
| Low-middle SDI | 65-69 years | 2.6 | 2.5 | 2.6 | Male |
| Low-middle SDI | 70-74 years | 2.4 | 2.4 | 2.5 | Male |
| Low-middle SDI | 75-79 years | 2.2 | 2.2 | 2.3 | Male |
| Low-middle SDI | 80-84 years | 2.2 | 2.1 | 2.3 | Male |
| Low-middle SDI | 85-89 years | 2.5 | 2.4 | 2.7 | Male |
| Low-middle SDI | 90-94 years | 2.9 | 2.7 | 3.1 | Male |
| Low-middle SDI | 95+ years | 3.8 | 3.7 | 4.0 | Male |
| Middle SDI | 20-24 years | 3.4 | 3.4 | 3.5 | Male |
| Middle SDI | 25-29 years | 3.1 | 3.0 | 3.3 | Male |
| Middle SDI | 30-34 years | 2.6 | 2.5 | 2.8 | Male |
| Middle SDI | 35-39 years | 2.4 | 2.3 | 2.5 | Male |
| Middle SDI | 40-44 years | 2.3 | 2.3 | 2.3 | Male |
| Middle SDI | 45-49 years | 2.2 | 2.2 | 2.3 | Male |
| Middle SDI | 50-54 years | 2.1 | 2.0 | 2.1 | Male |
| Middle SDI | 55-59 years | 2.1 | 2.1 | 2.2 | Male |
| Middle SDI | 60-64 years | 2.1 | 2.1 | 2.2 | Male |
| Middle SDI | 65-69 years | 2.0 | 1.9 | 2.0 | Male |
| Middle SDI | 70-74 years | 2.0 | 2.0 | 2.1 | Male |
| Middle SDI | 75-79 years | 2.1 | 2.1 | 2.1 | Male |
| Middle SDI | 80-84 years | 2.5 | 2.5 | 2.6 | Male |
| Middle SDI | 85-89 years | 2.5 | 2.5 | 2.6 | Male |
| Middle SDI | 90-94 years | 2.5 | 2.4 | 2.6 | Male |
| Middle SDI | 95+ years | 2.4 | 2.2 | 2.6 | Male |
| Global | 20-24 years | 2.3 | 2.2 | 2.3 | Female |
| Global | 25-29 years | 2.0 | 2.0 | 2.1 | Female |
| Global | 30-34 years | 1.6 | 1.5 | 1.8 | Female |
| Global | 35-39 years | 1.4 | 1.3 | 1.5 | Female |
| Global | 40-44 years | 1.2 | 1.1 | 1.3 | Female |
| Global | 45-49 years | 1.0 | 0.9 | 1.1 | Female |
| Global | 50-54 years | 1.0 | 0.9 | 1.1 | Female |
| Global | 55-59 years | 1.0 | 1.0 | 1.1 | Female |
| Global | 60-64 years | 1.0 | 0.9 | 1.1 | Female |
| Global | 65-69 years | 0.8 | 0.7 | 0.9 | Female |
| Global | 70-74 years | 0.8 | 0.7 | 0.8 | Female |
| Global | 75-79 years | 0.7 | 0.6 | 0.8 | Female |
| Global | 80-84 years | 0.6 | 0.5 | 0.7 | Female |
| Global | 85-89 years | 0.6 | 0.5 | 0.7 | Female |
| Global | 90-94 years | 0.6 | 0.5 | 0.7 | Female |
| Global | 95+ years | 0.5 | 0.5 | 0.6 | Female |
| High SDI | 20-24 years | 1.9 | 1.8 | 2.0 | Female |
| High SDI | 25-29 years | 1.5 | 1.5 | 1.6 | Female |
| High SDI | 30-34 years | 1.3 | 1.3 | 1.4 | Female |
| High SDI | 35-39 years | 1.2 | 1.2 | 1.2 | Female |
| High SDI | 40-44 years | 1.0 | 0.9 | 1.0 | Female |
| High SDI | 45-49 years | 0.9 | 0.8 | 0.9 | Female |
| High SDI | 50-54 years | 0.8 | 0.8 | 0.9 | Female |
| High SDI | 55-59 years | 0.8 | 0.8 | 0.9 | Female |
| High SDI | 60-64 years | 0.9 | 0.8 | 0.9 | Female |
| High SDI | 65-69 years | 0.9 | 0.9 | 1.0 | Female |
| High SDI | 70-74 years | 0.9 | 0.9 | 0.9 | Female |
| High SDI | 75-79 years | 0.9 | 0.8 | 0.9 | Female |
| High SDI | 80-84 years | 0.7 | 0.7 | 0.8 | Female |
| High SDI | 85-89 years | 0.7 | 0.6 | 0.7 | Female |
| High SDI | 90-94 years | 0.6 | 0.5 | 0.7 | Female |
| High SDI | 95+ years | 0.4 | 0.3 | 0.5 | Female |
| High-middle SDI | 20-24 years | 3.1 | 2.9 | 3.3 | Female |
| High-middle SDI | 25-29 years | 2.5 | 2.4 | 2.7 | Female |
| High-middle SDI | 30-34 years | 1.6 | 1.4 | 1.9 | Female |
| High-middle SDI | 35-39 years | 1.2 | 0.9 | 1.4 | Female |
| High-middle SDI | 40-44 years | 0.7 | 0.4 | 0.9 | Female |
| High-middle SDI | 45-49 years | 0.5 | 0.3 | 0.6 | Female |
| High-middle SDI | 50-54 years | 0.3 | 0.2 | 0.5 | Female |
| High-middle SDI | 55-59 years | 0.5 | 0.3 | 0.6 | Female |
| High-middle SDI | 60-64 years | 0.5 | 0.3 | 0.6 | Female |
| High-middle SDI | 65-69 years | 0.3 | 0.2 | 0.4 | Female |
| High-middle SDI | 70-74 years | 0.5 | 0.4 | 0.7 | Female |
| High-middle SDI | 75-79 years | 0.6 | 0.5 | 0.7 | Female |
| High-middle SDI | 80-84 years | 0.6 | 0.5 | 0.7 | Female |
| High-middle SDI | 85-89 years | 0.5 | 0.4 | 0.6 | Female |
| High-middle SDI | 90-94 years | 0.5 | 0.4 | 0.6 | Female |
| High-middle SDI | 95+ years | 0.4 | 0.4 | 0.5 | Female |
| Low SDI | 20-24 years | 1.7 | 1.6 | 1.8 | Female |
| Low SDI | 25-29 years | 2.0 | 2.0 | 2.1 | Female |
| Low SDI | 30-34 years | 2.2 | 2.1 | 2.2 | Female |
| Low SDI | 35-39 years | 2.1 | 2.0 | 2.2 | Female |
| Low SDI | 40-44 years | 2.0 | 1.9 | 2.1 | Female |
| Low SDI | 45-49 years | 1.6 | 1.5 | 1.7 | Female |
| Low SDI | 50-54 years | 1.6 | 1.5 | 1.7 | Female |
| Low SDI | 55-59 years | 1.6 | 1.6 | 1.6 | Female |
| Low SDI | 60-64 years | 1.5 | 1.5 | 1.6 | Female |
| Low SDI | 65-69 years | 1.4 | 1.4 | 1.4 | Female |
| Low SDI | 70-74 years | 1.5 | 1.4 | 1.5 | Female |
| Low SDI | 75-79 years | 1.4 | 1.4 | 1.5 | Female |
| Low SDI | 80-84 years | 1.4 | 1.4 | 1.5 | Female |
| Low SDI | 85-89 years | 1.5 | 1.4 | 1.6 | Female |
| Low SDI | 90-94 years | 1.6 | 1.4 | 1.8 | Female |
| Low SDI | 95+ years | 1.5 | 1.3 | 1.7 | Female |
| Low-middle SDI | 20-24 years | 2.6 | 2.5 | 2.7 | Female |
| Low-middle SDI | 25-29 years | 2.6 | 2.5 | 2.7 | Female |
| Low-middle SDI | 30-34 years | 2.5 | 2.3 | 2.6 | Female |
| Low-middle SDI | 35-39 years | 2.3 | 2.2 | 2.4 | Female |
| Low-middle SDI | 40-44 years | 2.4 | 2.4 | 2.5 | Female |
| Low-middle SDI | 45-49 years | 2.0 | 2.0 | 2.1 | Female |
| Low-middle SDI | 50-54 years | 2.3 | 2.2 | 2.4 | Female |
| Low-middle SDI | 55-59 years | 2.5 | 2.4 | 2.6 | Female |
| Low-middle SDI | 60-64 years | 2.5 | 2.4 | 2.6 | Female |
| Low-middle SDI | 65-69 years | 2.2 | 2.1 | 2.4 | Female |
| Low-middle SDI | 70-74 years | 1.8 | 1.7 | 1.9 | Female |
| Low-middle SDI | 75-79 years | 1.6 | 1.5 | 1.6 | Female |
| Low-middle SDI | 80-84 years | 1.6 | 1.5 | 1.6 | Female |
| Low-middle SDI | 85-89 years | 1.6 | 1.6 | 1.7 | Female |
| Low-middle SDI | 90-94 years | 1.7 | 1.6 | 1.7 | Female |
| Low-middle SDI | 95+ years | 2.2 | 2.0 | 2.3 | Female |
| Middle SDI | 20-24 years | 3.1 | 3.0 | 3.2 | Female |
| Middle SDI | 25-29 years | 2.8 | 2.6 | 2.9 | Female |
| Middle SDI | 30-34 years | 2.3 | 2.1 | 2.5 | Female |
| Middle SDI | 35-39 years | 2.1 | 1.9 | 2.3 | Female |
| Middle SDI | 40-44 years | 2.0 | 1.9 | 2.2 | Female |
| Middle SDI | 45-49 years | 1.8 | 1.7 | 1.8 | Female |
| Middle SDI | 50-54 years | 1.9 | 1.8 | 2.0 | Female |
| Middle SDI | 55-59 years | 2.0 | 1.9 | 2.0 | Female |
| Middle SDI | 60-64 years | 2.0 | 2.0 | 2.1 | Female |
| Middle SDI | 65-69 years | 2.1 | 2.0 | 2.1 | Female |
| Middle SDI | 70-74 years | 2.1 | 2.0 | 2.2 | Female |
| Middle SDI | 75-79 years | 2.0 | 2.0 | 2.1 | Female |
| Middle SDI | 80-84 years | 2.0 | 1.9 | 2.0 | Female |
| Middle SDI | 85-89 years | 1.7 | 1.7 | 1.8 | Female |
| Middle SDI | 90-94 years | 1.5 | 1.5 | 1.6 | Female |
| Middle SDI | 95+ years | 1.6 | 1.6 | 1.6 | Female |

***Footnote***: EAPC: Estimated annual percentage change; SDI: Socio-demographic index.

## Supplementary Table S3. Predicted Years Lived with Disability Rates (per 100,000) for Low Back Pain Attributable to High Body Mass Index in Global and Five Socio-Demographic Index Regions for Both Males and Females from 2022 to 2050.

| year | val | lower | upper | location_name | sex_name |
| --- | --- | --- | --- | --- | --- |
| 2022 | 72.2 | 72.0 | 72.4 | Global | Male |
| 2023 | 73.1 | 72.6 | 73.6 | Global | Male |
| 2024 | 74.0 | 73.0 | 74.9 | Global | Male |
| 2025 | 74.8 | 73.4 | 76.4 | Global | Male |
| 2026 | 75.7 | 73.6 | 77.8 | Global | Male |
| 2027 | 76.6 | 73.8 | 79.4 | Global | Male |
| 2028 | 77.4 | 74.0 | 81.1 | Global | Male |
| 2029 | 78.3 | 74.1 | 82.8 | Global | Male |
| 2030 | 79.2 | 74.1 | 84.6 | Global | Male |
| 2031 | 80.0 | 74.1 | 86.4 | Global | Male |
| 2032 | 80.9 | 74.1 | 88.3 | Global | Male |
| 2033 | 81.8 | 74.0 | 90.3 | Global | Male |
| 2034 | 82.6 | 73.9 | 92.4 | Global | Male |
| 2035 | 83.5 | 73.7 | 94.5 | Global | Male |
| 2036 | 84.3 | 73.5 | 96.8 | Global | Male |
| 2037 | 85.2 | 73.3 | 99.0 | Global | Male |
| 2038 | 86.0 | 73.0 | 101.4 | Global | Male |
| 2039 | 86.9 | 72.7 | 103.8 | Global | Male |
| 2040 | 87.7 | 72.4 | 106.3 | Global | Male |
| 2041 | 88.6 | 72.0 | 108.9 | Global | Male |
| 2042 | 89.4 | 71.6 | 111.6 | Global | Male |
| 2043 | 90.2 | 71.2 | 114.3 | Global | Male |
| 2044 | 91.1 | 70.8 | 117.1 | Global | Male |
| 2045 | 91.9 | 70.4 | 120.0 | Global | Male |
| 2046 | 92.7 | 69.9 | 123.0 | Global | Male |
| 2047 | 93.6 | 69.4 | 126.1 | Global | Male |
| 2048 | 94.4 | 68.9 | 129.2 | Global | Male |
| 2049 | 95.2 | 68.4 | 132.5 | Global | Male |
| 2050 | 96.0 | 67.9 | 135.8 | Global | Male |
| 2022 | 143.9 | 143.4 | 144.4 | Global | Female |
| 2023 | 146.9 | 145.7 | 148.1 | Global | Female |
| 2024 | 149.9 | 147.9 | 152.0 | Global | Female |
| 2025 | 153.1 | 150.1 | 156.1 | Global | Female |
| 2026 | 156.3 | 152.1 | 160.5 | Global | Female |
| 2027 | 159.5 | 154.1 | 165.1 | Global | Female |
| 2028 | 162.9 | 156.1 | 170.0 | Global | Female |
| 2029 | 166.3 | 157.9 | 175.0 | Global | Female |
| 2030 | 169.7 | 159.7 | 180.4 | Global | Female |
| 2031 | 173.3 | 161.4 | 186.0 | Global | Female |
| 2032 | 176.9 | 163.1 | 191.8 | Global | Female |
| 2033 | 180.6 | 164.7 | 198.0 | Global | Female |
| 2034 | 184.3 | 166.3 | 204.4 | Global | Female |
| 2035 | 188.2 | 167.8 | 211.1 | Global | Female |
| 2036 | 192.1 | 169.2 | 218.1 | Global | Female |
| 2037 | 196.1 | 170.6 | 225.5 | Global | Female |
| 2038 | 200.2 | 171.9 | 233.2 | Global | Female |
| 2039 | 204.4 | 173.2 | 241.2 | Global | Female |
| 2040 | 208.7 | 174.4 | 249.6 | Global | Female |
| 2041 | 213.0 | 175.6 | 258.4 | Global | Female |
| 2042 | 217.5 | 176.7 | 267.6 | Global | Female |
| 2043 | 222.0 | 177.8 | 277.2 | Global | Female |
| 2044 | 226.6 | 178.8 | 287.3 | Global | Female |
| 2045 | 231.4 | 179.8 | 297.8 | Global | Female |
| 2046 | 236.2 | 180.7 | 308.8 | Global | Female |
| 2047 | 241.1 | 181.5 | 320.3 | Global | Female |
| 2048 | 246.2 | 182.3 | 332.3 | Global | Female |
| 2049 | 251.3 | 183.1 | 344.9 | Global | Female |
| 2050 | 256.5 | 183.8 | 358.1 | Global | Female |
| 2022 | 108.1 | 107.8 | 108.4 | Global | Both |
| 2023 | 110.2 | 109.4 | 111.0 | Global | Both |
| 2024 | 112.5 | 110.9 | 114.0 | Global | Both |
| 2025 | 114.7 | 112.2 | 117.3 | Global | Both |
| 2026 | 117.1 | 113.4 | 120.9 | Global | Both |
| 2027 | 119.5 | 114.4 | 124.8 | Global | Both |
| 2028 | 121.9 | 115.2 | 129.0 | Global | Both |
| 2029 | 124.4 | 116.0 | 133.4 | Global | Both |
| 2030 | 127.0 | 116.6 | 138.2 | Global | Both |
| 2031 | 129.6 | 117.2 | 143.3 | Global | Both |
| 2032 | 132.2 | 117.6 | 148.7 | Global | Both |
| 2033 | 134.9 | 117.9 | 154.4 | Global | Both |
| 2034 | 137.7 | 118.1 | 160.5 | Global | Both |
| 2035 | 140.5 | 118.2 | 167.0 | Global | Both |
| 2036 | 143.4 | 118.3 | 173.8 | Global | Both |
| 2037 | 146.3 | 118.2 | 181.1 | Global | Both |
| 2038 | 149.3 | 118.1 | 188.8 | Global | Both |
| 2039 | 152.4 | 117.9 | 197.0 | Global | Both |
| 2040 | 155.5 | 117.6 | 205.6 | Global | Both |
| 2041 | 158.7 | 117.2 | 214.8 | Global | Both |
| 2042 | 161.9 | 116.8 | 224.5 | Global | Both |
| 2043 | 165.3 | 116.3 | 234.8 | Global | Both |
| 2044 | 168.6 | 115.7 | 245.7 | Global | Both |
| 2045 | 172.1 | 115.1 | 257.3 | Global | Both |
| 2046 | 175.6 | 114.4 | 269.6 | Global | Both |
| 2047 | 179.2 | 113.6 | 282.6 | Global | Both |
| 2048 | 182.9 | 112.8 | 296.4 | Global | Both |
| 2049 | 186.6 | 112.0 | 311.1 | Global | Both |
| 2050 | 190.5 | 111.1 | 326.6 | Global | Both |
| 2022 | 105.5 | 105.1 | 105.9 | High-middle SDI | Male |
| 2023 | 107.2 | 106.2 | 108.3 | High-middle SDI | Male |
| 2024 | 108.9 | 107.1 | 110.8 | High-middle SDI | Male |
| 2025 | 110.7 | 107.8 | 113.6 | High-middle SDI | Male |
| 2026 | 112.4 | 108.5 | 116.5 | High-middle SDI | Male |
| 2027 | 114.1 | 109.0 | 119.5 | High-middle SDI | Male |
| 2028 | 115.9 | 109.4 | 122.7 | High-middle SDI | Male |
| 2029 | 117.6 | 109.7 | 126.1 | High-middle SDI | Male |
| 2030 | 119.4 | 109.9 | 129.7 | High-middle SDI | Male |
| 2031 | 121.2 | 110.0 | 133.4 | High-middle SDI | Male |
| 2032 | 122.9 | 110.1 | 137.3 | High-middle SDI | Male |
| 2033 | 124.7 | 110.1 | 141.3 | High-middle SDI | Male |
| 2034 | 126.5 | 110.0 | 145.5 | High-middle SDI | Male |
| 2035 | 128.3 | 109.8 | 149.9 | High-middle SDI | Male |
| 2036 | 130.1 | 109.6 | 154.5 | High-middle SDI | Male |
| 2037 | 131.9 | 109.3 | 159.2 | High-middle SDI | Male |
| 2038 | 133.7 | 108.9 | 164.1 | High-middle SDI | Male |
| 2039 | 135.5 | 108.5 | 169.3 | High-middle SDI | Male |
| 2040 | 137.3 | 108.0 | 174.6 | High-middle SDI | Male |
| 2041 | 139.1 | 107.5 | 180.1 | High-middle SDI | Male |
| 2042 | 141.0 | 106.9 | 185.8 | High-middle SDI | Male |
| 2043 | 142.8 | 106.3 | 191.8 | High-middle SDI | Male |
| 2044 | 144.6 | 105.6 | 198.0 | High-middle SDI | Male |
| 2045 | 146.4 | 104.9 | 204.4 | High-middle SDI | Male |
| 2046 | 148.3 | 104.2 | 211.0 | High-middle SDI | Male |
| 2047 | 150.1 | 103.4 | 217.9 | High-middle SDI | Male |
| 2048 | 151.9 | 102.6 | 225.0 | High-middle SDI | Male |
| 2049 | 153.8 | 101.8 | 232.4 | High-middle SDI | Male |
| 2050 | 155.6 | 100.9 | 240.0 | High-middle SDI | Male |
| 2022 | 221.8 | 220.9 | 222.7 | High-middle SDI | Female |
| 2023 | 227.6 | 225.2 | 230.0 | High-middle SDI | Female |
| 2024 | 233.6 | 229.1 | 238.1 | High-middle SDI | Female |
| 2025 | 239.7 | 232.8 | 246.9 | High-middle SDI | Female |
| 2026 | 246.1 | 236.2 | 256.4 | High-middle SDI | Female |
| 2027 | 252.6 | 239.3 | 266.6 | High-middle SDI | Female |
| 2028 | 259.3 | 242.2 | 277.6 | High-middle SDI | Female |
| 2029 | 266.1 | 244.8 | 289.3 | High-middle SDI | Female |
| 2030 | 273.2 | 247.3 | 301.8 | High-middle SDI | Female |
| 2031 | 280.4 | 249.5 | 315.2 | High-middle SDI | Female |
| 2032 | 287.8 | 251.5 | 329.4 | High-middle SDI | Female |
| 2033 | 295.4 | 253.3 | 344.6 | High-middle SDI | Female |
| 2034 | 303.3 | 255.0 | 360.7 | High-middle SDI | Female |
| 2035 | 311.3 | 256.4 | 377.9 | High-middle SDI | Female |
| 2036 | 319.5 | 257.7 | 396.2 | High-middle SDI | Female |
| 2037 | 328.0 | 258.7 | 415.8 | High-middle SDI | Female |
| 2038 | 336.7 | 259.6 | 436.5 | High-middle SDI | Female |
| 2039 | 345.6 | 260.3 | 458.7 | High-middle SDI | Female |
| 2040 | 354.7 | 260.9 | 482.2 | High-middle SDI | Female |
| 2041 | 364.1 | 261.3 | 507.3 | High-middle SDI | Female |
| 2042 | 373.7 | 261.5 | 534.1 | High-middle SDI | Female |
| 2043 | 383.6 | 261.6 | 562.6 | High-middle SDI | Female |
| 2044 | 393.8 | 261.5 | 592.9 | High-middle SDI | Female |
| 2045 | 404.2 | 261.2 | 625.3 | High-middle SDI | Female |
| 2046 | 414.9 | 260.8 | 659.8 | High-middle SDI | Female |
| 2047 | 425.8 | 260.3 | 696.7 | High-middle SDI | Female |
| 2048 | 437.1 | 259.6 | 735.9 | High-middle SDI | Female |
| 2049 | 448.7 | 258.8 | 777.8 | High-middle SDI | Female |
| 2050 | 460.6 | 257.9 | 822.6 | High-middle SDI | Female |
| 2022 | 163.7 | 163.2 | 164.1 | High-middle SDI | Both |
| 2023 | 167.6 | 166.2 | 169.0 | High-middle SDI | Both |
| 2024 | 171.7 | 168.8 | 174.7 | High-middle SDI | Both |
| 2025 | 176.1 | 171.1 | 181.2 | High-middle SDI | Both |
| 2026 | 180.6 | 173.1 | 188.4 | High-middle SDI | Both |
| 2027 | 185.3 | 174.6 | 196.6 | High-middle SDI | Both |
| 2028 | 190.1 | 175.8 | 205.6 | High-middle SDI | Both |
| 2029 | 195.2 | 176.6 | 215.6 | High-middle SDI | Both |
| 2030 | 200.3 | 177.1 | 226.6 | High-middle SDI | Both |
| 2031 | 205.7 | 177.2 | 238.6 | High-middle SDI | Both |
| 2032 | 211.1 | 177.0 | 251.8 | High-middle SDI | Both |
| 2033 | 216.8 | 176.5 | 266.2 | High-middle SDI | Both |
| 2034 | 222.6 | 175.7 | 281.9 | High-middle SDI | Both |
| 2035 | 228.5 | 174.7 | 299.0 | High-middle SDI | Both |
| 2036 | 234.7 | 173.3 | 317.7 | High-middle SDI | Both |
| 2037 | 241.0 | 171.8 | 338.0 | High-middle SDI | Both |
| 2038 | 247.4 | 170.0 | 360.2 | High-middle SDI | Both |
| 2039 | 254.1 | 168.0 | 384.3 | High-middle SDI | Both |
| 2040 | 260.9 | 165.8 | 410.6 | High-middle SDI | Both |
| 2041 | 267.9 | 163.4 | 439.3 | High-middle SDI | Both |
| 2042 | 275.1 | 160.8 | 470.5 | High-middle SDI | Both |
| 2043 | 282.5 | 158.2 | 504.6 | High-middle SDI | Both |
| 2044 | 290.1 | 155.3 | 541.7 | High-middle SDI | Both |
| 2045 | 297.9 | 152.4 | 582.2 | High-middle SDI | Both |
| 2046 | 305.9 | 149.3 | 626.5 | High-middle SDI | Both |
| 2047 | 314.1 | 146.2 | 674.8 | High-middle SDI | Both |
| 2048 | 322.5 | 143.0 | 727.7 | High-middle SDI | Both |
| 2049 | 331.2 | 139.7 | 785.5 | High-middle SDI | Both |
| 2050 | 340.1 | 136.3 | 848.7 | High-middle SDI | Both |
| 2022 | 185.5 | 185.0 | 186.1 | High SDI | Male |
| 2023 | 187.0 | 185.5 | 188.6 | High SDI | Male |
| 2024 | 188.4 | 185.4 | 191.5 | High SDI | Male |
| 2025 | 189.8 | 184.8 | 194.9 | High SDI | Male |
| 2026 | 191.0 | 183.7 | 198.7 | High SDI | Male |
| 2027 | 192.3 | 182.1 | 203.0 | High SDI | Male |
| 2028 | 193.5 | 180.2 | 207.7 | High SDI | Male |
| 2029 | 194.7 | 178.0 | 212.9 | High SDI | Male |
| 2030 | 195.9 | 175.4 | 218.7 | High SDI | Male |
| 2031 | 197.0 | 172.6 | 224.9 | High SDI | Male |
| 2032 | 198.2 | 169.6 | 231.6 | High SDI | Male |
| 2033 | 199.4 | 166.5 | 238.9 | High SDI | Male |
| 2034 | 200.6 | 163.1 | 246.7 | High SDI | Male |
| 2035 | 201.8 | 159.6 | 255.1 | High SDI | Male |
| 2036 | 203.0 | 156.0 | 264.1 | High SDI | Male |
| 2037 | 204.2 | 152.3 | 273.8 | High SDI | Male |
| 2038 | 205.4 | 148.5 | 284.1 | High SDI | Male |
| 2039 | 206.7 | 144.7 | 295.1 | High SDI | Male |
| 2040 | 207.9 | 140.8 | 306.9 | High SDI | Male |
| 2041 | 209.1 | 136.9 | 319.5 | High SDI | Male |
| 2042 | 210.4 | 133.0 | 332.8 | High SDI | Male |
| 2043 | 211.6 | 129.0 | 347.1 | High SDI | Male |
| 2044 | 212.9 | 125.1 | 362.4 | High SDI | Male |
| 2045 | 214.1 | 121.1 | 378.6 | High SDI | Male |
| 2046 | 215.4 | 117.2 | 395.9 | High SDI | Male |
| 2047 | 216.7 | 113.3 | 414.4 | High SDI | Male |
| 2048 | 218.0 | 109.5 | 434.1 | High SDI | Male |
| 2049 | 219.3 | 105.7 | 455.0 | High SDI | Male |
| 2050 | 220.6 | 101.9 | 477.4 | High SDI | Male |
| 2022 | 271.3 | 269.4 | 273.2 | High SDI | Female |
| 2023 | 275.0 | 271.8 | 278.3 | High SDI | Female |
| 2024 | 277.9 | 272.7 | 283.1 | High SDI | Female |
| 2025 | 281.2 | 274.0 | 288.7 | High SDI | Female |
| 2026 | 284.3 | 274.6 | 294.3 | High SDI | Female |
| 2027 | 287.5 | 275.3 | 300.3 | High SDI | Female |
| 2028 | 290.6 | 275.6 | 306.5 | High SDI | Female |
| 2029 | 293.8 | 275.8 | 313.0 | High SDI | Female |
| 2030 | 296.9 | 275.7 | 319.7 | High SDI | Female |
| 2031 | 300.1 | 275.6 | 326.7 | High SDI | Female |
| 2032 | 303.2 | 275.2 | 333.9 | High SDI | Female |
| 2033 | 306.3 | 274.8 | 341.4 | High SDI | Female |
| 2034 | 309.4 | 274.2 | 349.2 | High SDI | Female |
| 2035 | 312.5 | 273.4 | 357.1 | High SDI | Female |
| 2036 | 315.6 | 272.6 | 365.4 | High SDI | Female |
| 2037 | 318.7 | 271.6 | 373.9 | High SDI | Female |
| 2038 | 321.7 | 270.5 | 382.7 | High SDI | Female |
| 2039 | 324.8 | 269.3 | 391.7 | High SDI | Female |
| 2040 | 327.8 | 268.0 | 401.0 | High SDI | Female |
| 2041 | 330.9 | 266.6 | 410.6 | High SDI | Female |
| 2042 | 333.9 | 265.1 | 420.5 | High SDI | Female |
| 2043 | 336.9 | 263.6 | 430.6 | High SDI | Female |
| 2044 | 339.9 | 262.0 | 441.1 | High SDI | Female |
| 2045 | 342.9 | 260.2 | 451.8 | High SDI | Female |
| 2046 | 345.9 | 258.5 | 462.8 | High SDI | Female |
| 2047 | 348.8 | 256.6 | 474.2 | High SDI | Female |
| 2048 | 351.8 | 254.8 | 485.8 | High SDI | Female |
| 2049 | 354.7 | 252.8 | 497.7 | High SDI | Female |
| 2050 | 357.7 | 250.8 | 510.0 | High SDI | Female |
| 2022 | 229.9 | 228.7 | 231.1 | High SDI | Both |
| 2023 | 233.3 | 230.6 | 236.0 | High SDI | Both |
| 2024 | 236.7 | 232.1 | 241.3 | High SDI | Both |
| 2025 | 240.1 | 233.4 | 247.1 | High SDI | Both |
| 2026 | 243.7 | 234.5 | 253.2 | High SDI | Both |
| 2027 | 247.2 | 235.3 | 259.8 | High SDI | Both |
| 2028 | 250.9 | 235.9 | 266.8 | High SDI | Both |
| 2029 | 254.6 | 236.4 | 274.1 | High SDI | Both |
| 2030 | 258.3 | 236.6 | 281.9 | High SDI | Both |
| 2031 | 262.1 | 236.7 | 290.2 | High SDI | Both |
| 2032 | 265.9 | 236.6 | 298.8 | High SDI | Both |
| 2033 | 269.8 | 236.4 | 308.0 | High SDI | Both |
| 2034 | 273.8 | 236.0 | 317.6 | High SDI | Both |
| 2035 | 277.8 | 235.5 | 327.7 | High SDI | Both |
| 2036 | 281.9 | 234.8 | 338.4 | High SDI | Both |
| 2037 | 286.0 | 234.0 | 349.6 | High SDI | Both |
| 2038 | 290.2 | 233.1 | 361.3 | High SDI | Both |
| 2039 | 294.5 | 232.0 | 373.7 | High SDI | Both |
| 2040 | 298.8 | 230.9 | 386.7 | High SDI | Both |
| 2041 | 303.2 | 229.6 | 400.3 | High SDI | Both |
| 2042 | 307.6 | 228.2 | 414.7 | High SDI | Both |
| 2043 | 312.1 | 226.7 | 429.7 | High SDI | Both |
| 2044 | 316.7 | 225.1 | 445.5 | High SDI | Both |
| 2045 | 321.4 | 223.5 | 462.1 | High SDI | Both |
| 2046 | 326.1 | 221.7 | 479.5 | High SDI | Both |
| 2047 | 330.9 | 219.9 | 497.9 | High SDI | Both |
| 2048 | 335.7 | 218.0 | 517.1 | High SDI | Both |
| 2049 | 340.6 | 216.0 | 537.3 | High SDI | Both |
| 2050 | 345.6 | 213.9 | 558.5 | High SDI | Both |
| 2022 | 36.5 | 34.1 | 39.0 | Low-middle SDI | Male |
| 2023 | 36.5 | 33.1 | 40.2 | Low-middle SDI | Male |
| 2024 | 36.5 | 32.4 | 41.0 | Low-middle SDI | Male |
| 2025 | 36.5 | 31.8 | 41.8 | Low-middle SDI | Male |
| 2026 | 36.5 | 31.3 | 42.5 | Low-middle SDI | Male |
| 2027 | 36.5 | 30.9 | 43.1 | Low-middle SDI | Male |
| 2028 | 36.5 | 30.5 | 43.7 | Low-middle SDI | Male |
| 2029 | 36.5 | 30.1 | 44.2 | Low-middle SDI | Male |
| 2030 | 36.5 | 29.8 | 44.7 | Low-middle SDI | Male |
| 2031 | 36.5 | 29.4 | 45.2 | Low-middle SDI | Male |
| 2032 | 36.5 | 29.1 | 45.7 | Low-middle SDI | Male |
| 2033 | 36.5 | 28.8 | 46.2 | Low-middle SDI | Male |
| 2034 | 36.5 | 28.6 | 46.6 | Low-middle SDI | Male |
| 2035 | 36.5 | 28.3 | 47.0 | Low-middle SDI | Male |
| 2036 | 36.5 | 28.0 | 47.5 | Low-middle SDI | Male |
| 2037 | 36.5 | 27.8 | 47.9 | Low-middle SDI | Male |
| 2038 | 36.5 | 27.6 | 48.3 | Low-middle SDI | Male |
| 2039 | 36.5 | 27.3 | 48.7 | Low-middle SDI | Male |
| 2040 | 36.5 | 27.1 | 49.1 | Low-middle SDI | Male |
| 2041 | 36.5 | 26.9 | 49.4 | Low-middle SDI | Male |
| 2042 | 36.5 | 26.7 | 49.8 | Low-middle SDI | Male |
| 2043 | 36.5 | 26.5 | 50.2 | Low-middle SDI | Male |
| 2044 | 36.5 | 26.3 | 50.5 | Low-middle SDI | Male |
| 2045 | 36.5 | 26.2 | 50.9 | Low-middle SDI | Male |
| 2046 | 36.5 | 26.0 | 51.2 | Low-middle SDI | Male |
| 2047 | 36.5 | 25.8 | 51.6 | Low-middle SDI | Male |
| 2048 | 36.5 | 25.6 | 51.9 | Low-middle SDI | Male |
| 2049 | 36.5 | 25.5 | 52.3 | Low-middle SDI | Male |
| 2050 | 36.5 | 25.3 | 52.6 | Low-middle SDI | Male |
| 2022 | 93.3 | 93.0 | 93.5 | Low-middle SDI | Female |
| 2023 | 94.5 | 93.6 | 95.4 | Low-middle SDI | Female |
| 2024 | 95.5 | 93.6 | 97.5 | Low-middle SDI | Female |
| 2025 | 96.8 | 93.6 | 100.1 | Low-middle SDI | Female |
| 2026 | 98.2 | 93.6 | 103.1 | Low-middle SDI | Female |
| 2027 | 99.7 | 93.5 | 106.3 | Low-middle SDI | Female |
| 2028 | 100.9 | 93.0 | 109.4 | Low-middle SDI | Female |
| 2029 | 101.8 | 92.1 | 112.5 | Low-middle SDI | Female |
| 2030 | 102.5 | 90.8 | 115.8 | Low-middle SDI | Female |
| 2031 | 103.4 | 89.4 | 119.5 | Low-middle SDI | Female |
| 2032 | 104.4 | 88.0 | 123.8 | Low-middle SDI | Female |
| 2033 | 105.4 | 86.6 | 128.3 | Low-middle SDI | Female |
| 2034 | 106.4 | 85.0 | 133.1 | Low-middle SDI | Female |
| 2035 | 107.1 | 83.2 | 137.8 | Low-middle SDI | Female |
| 2036 | 107.8 | 81.3 | 142.8 | Low-middle SDI | Female |
| 2037 | 108.4 | 79.3 | 148.2 | Low-middle SDI | Female |
| 2038 | 109.2 | 77.4 | 154.1 | Low-middle SDI | Female |
| 2039 | 110.0 | 75.4 | 160.5 | Low-middle SDI | Female |
| 2040 | 110.8 | 73.5 | 167.1 | Low-middle SDI | Female |
| 2041 | 111.5 | 71.5 | 174.0 | Low-middle SDI | Female |
| 2042 | 112.1 | 69.4 | 181.1 | Low-middle SDI | Female |
| 2043 | 112.7 | 67.3 | 188.6 | Low-middle SDI | Female |
| 2044 | 113.3 | 65.3 | 196.7 | Low-middle SDI | Female |
| 2045 | 114.0 | 63.3 | 205.2 | Low-middle SDI | Female |
| 2046 | 114.7 | 61.4 | 214.3 | Low-middle SDI | Female |
| 2047 | 115.3 | 59.4 | 223.7 | Low-middle SDI | Female |
| 2048 | 115.9 | 57.5 | 233.5 | Low-middle SDI | Female |
| 2049 | 116.4 | 55.6 | 243.7 | Low-middle SDI | Female |
| 2050 | 117.0 | 53.8 | 254.5 | Low-middle SDI | Female |
| 2022 | 65.2 | 65.0 | 65.4 | Low-middle SDI | Both |
| 2023 | 66.4 | 65.7 | 67.1 | Low-middle SDI | Both |
| 2024 | 67.6 | 66.3 | 69.1 | Low-middle SDI | Both |
| 2025 | 68.8 | 66.6 | 71.2 | Low-middle SDI | Both |
| 2026 | 70.0 | 66.8 | 73.4 | Low-middle SDI | Both |
| 2027 | 71.2 | 66.9 | 75.9 | Low-middle SDI | Both |
| 2028 | 72.5 | 66.8 | 78.6 | Low-middle SDI | Both |
| 2029 | 73.7 | 66.7 | 81.4 | Low-middle SDI | Both |
| 2030 | 74.9 | 66.5 | 84.4 | Low-middle SDI | Both |
| 2031 | 76.1 | 66.2 | 87.6 | Low-middle SDI | Both |
| 2032 | 77.4 | 65.8 | 91.0 | Low-middle SDI | Both |
| 2033 | 78.6 | 65.4 | 94.5 | Low-middle SDI | Both |
| 2034 | 79.9 | 64.9 | 98.3 | Low-middle SDI | Both |
| 2035 | 81.1 | 64.3 | 102.3 | Low-middle SDI | Both |
| 2036 | 82.4 | 63.7 | 106.5 | Low-middle SDI | Both |
| 2037 | 83.6 | 63.0 | 111.0 | Low-middle SDI | Both |
| 2038 | 84.9 | 62.3 | 115.7 | Low-middle SDI | Both |
| 2039 | 86.2 | 61.6 | 120.6 | Low-middle SDI | Both |
| 2040 | 87.4 | 60.8 | 125.8 | Low-middle SDI | Both |
| 2041 | 88.7 | 59.9 | 131.3 | Low-middle SDI | Both |
| 2042 | 90.0 | 59.1 | 137.1 | Low-middle SDI | Both |
| 2043 | 91.3 | 58.2 | 143.2 | Low-middle SDI | Both |
| 2044 | 92.6 | 57.3 | 149.5 | Low-middle SDI | Both |
| 2045 | 93.9 | 56.4 | 156.3 | Low-middle SDI | Both |
| 2046 | 95.2 | 55.4 | 163.3 | Low-middle SDI | Both |
| 2047 | 96.5 | 54.5 | 170.7 | Low-middle SDI | Both |
| 2048 | 97.8 | 53.5 | 178.6 | Low-middle SDI | Both |
| 2049 | 99.1 | 52.5 | 186.8 | Low-middle SDI | Both |
| 2050 | 100.4 | 51.6 | 195.4 | Low-middle SDI | Both |
| 2022 | 20.3 | 20.2 | 20.3 | Low SDI | Male |
| 2023 | 21.0 | 20.8 | 21.1 | Low SDI | Male |
| 2024 | 21.7 | 21.4 | 22.0 | Low SDI | Male |
| 2025 | 22.5 | 22.1 | 22.9 | Low SDI | Male |
| 2026 | 23.3 | 22.7 | 23.9 | Low SDI | Male |
| 2027 | 24.1 | 23.3 | 24.9 | Low SDI | Male |
| 2028 | 24.9 | 23.9 | 26.0 | Low SDI | Male |
| 2029 | 25.8 | 24.5 | 27.1 | Low SDI | Male |
| 2030 | 26.7 | 25.1 | 28.4 | Low SDI | Male |
| 2031 | 27.6 | 25.7 | 29.7 | Low SDI | Male |
| 2032 | 28.6 | 26.3 | 31.1 | Low SDI | Male |
| 2033 | 29.6 | 26.9 | 32.5 | Low SDI | Male |
| 2034 | 30.6 | 27.5 | 34.1 | Low SDI | Male |
| 2035 | 31.7 | 28.1 | 35.7 | Low SDI | Male |
| 2036 | 32.8 | 28.7 | 37.4 | Low SDI | Male |
| 2037 | 33.9 | 29.3 | 39.3 | Low SDI | Male |
| 2038 | 35.1 | 29.9 | 41.2 | Low SDI | Male |
| 2039 | 36.3 | 30.5 | 43.2 | Low SDI | Male |
| 2040 | 37.6 | 31.1 | 45.4 | Low SDI | Male |
| 2041 | 38.9 | 31.8 | 47.7 | Low SDI | Male |
| 2042 | 40.3 | 32.4 | 50.1 | Low SDI | Male |
| 2043 | 41.7 | 33.0 | 52.7 | Low SDI | Male |
| 2044 | 43.1 | 33.6 | 55.5 | Low SDI | Male |
| 2045 | 44.7 | 34.2 | 58.4 | Low SDI | Male |
| 2046 | 46.2 | 34.8 | 61.4 | Low SDI | Male |
| 2047 | 47.8 | 35.4 | 64.7 | Low SDI | Male |
| 2048 | 49.5 | 36.0 | 68.1 | Low SDI | Male |
| 2049 | 51.2 | 36.6 | 71.8 | Low SDI | Male |
| 2050 | 53.0 | 37.2 | 75.6 | Low SDI | Male |
| 2022 | 45.3 | 45.2 | 45.4 | Low SDI | Female |
| 2023 | 46.2 | 45.9 | 46.6 | Low SDI | Female |
| 2024 | 47.2 | 46.5 | 48.0 | Low SDI | Female |
| 2025 | 48.2 | 46.9 | 49.6 | Low SDI | Female |
| 2026 | 49.2 | 47.2 | 51.3 | Low SDI | Female |
| 2027 | 50.3 | 47.5 | 53.2 | Low SDI | Female |
| 2028 | 51.3 | 47.7 | 55.2 | Low SDI | Female |
| 2029 | 52.4 | 47.8 | 57.5 | Low SDI | Female |
| 2030 | 53.5 | 47.8 | 59.8 | Low SDI | Female |
| 2031 | 54.6 | 47.8 | 62.4 | Low SDI | Female |
| 2032 | 55.8 | 47.7 | 65.2 | Low SDI | Female |
| 2033 | 56.9 | 47.6 | 68.1 | Low SDI | Female |
| 2034 | 58.1 | 47.4 | 71.2 | Low SDI | Female |
| 2035 | 59.4 | 47.2 | 74.6 | Low SDI | Female |
| 2036 | 60.6 | 47.0 | 78.2 | Low SDI | Female |
| 2037 | 61.9 | 46.6 | 82.1 | Low SDI | Female |
| 2038 | 63.2 | 46.3 | 86.2 | Low SDI | Female |
| 2039 | 64.5 | 45.9 | 90.7 | Low SDI | Female |
| 2040 | 65.9 | 45.5 | 95.4 | Low SDI | Female |
| 2041 | 67.3 | 45.0 | 100.4 | Low SDI | Female |
| 2042 | 68.7 | 44.5 | 105.9 | Low SDI | Female |
| 2043 | 70.1 | 44.0 | 111.7 | Low SDI | Female |
| 2044 | 71.6 | 43.5 | 117.9 | Low SDI | Female |
| 2045 | 73.1 | 42.9 | 124.5 | Low SDI | Female |
| 2046 | 74.6 | 42.3 | 131.6 | Low SDI | Female |
| 2047 | 76.2 | 41.7 | 139.3 | Low SDI | Female |
| 2048 | 77.8 | 41.1 | 147.4 | Low SDI | Female |
| 2049 | 79.4 | 40.4 | 156.2 | Low SDI | Female |
| 2050 | 81.1 | 39.7 | 165.6 | Low SDI | Female |
| 2022 | 32.8 | 32.7 | 32.8 | Low SDI | Both |
| 2023 | 33.6 | 33.4 | 33.8 | Low SDI | Both |
| 2024 | 34.5 | 34.1 | 35.0 | Low SDI | Both |
| 2025 | 35.4 | 34.7 | 36.2 | Low SDI | Both |
| 2026 | 36.4 | 35.3 | 37.5 | Low SDI | Both |
| 2027 | 37.3 | 35.8 | 38.9 | Low SDI | Both |
| 2028 | 38.3 | 36.4 | 40.4 | Low SDI | Both |
| 2029 | 39.3 | 36.9 | 42.0 | Low SDI | Both |
| 2030 | 40.4 | 37.3 | 43.6 | Low SDI | Both |
| 2031 | 41.4 | 37.8 | 45.4 | Low SDI | Both |
| 2032 | 42.5 | 38.2 | 47.3 | Low SDI | Both |
| 2033 | 43.7 | 38.6 | 49.3 | Low SDI | Both |
| 2034 | 44.8 | 39.0 | 51.5 | Low SDI | Both |
| 2035 | 46.0 | 39.4 | 53.7 | Low SDI | Both |
| 2036 | 47.2 | 39.7 | 56.1 | Low SDI | Both |
| 2037 | 48.5 | 40.0 | 58.7 | Low SDI | Both |
| 2038 | 49.7 | 40.3 | 61.4 | Low SDI | Both |
| 2039 | 51.0 | 40.6 | 64.2 | Low SDI | Both |
| 2040 | 52.4 | 40.8 | 67.2 | Low SDI | Both |
| 2041 | 53.8 | 41.1 | 70.4 | Low SDI | Both |
| 2042 | 55.2 | 41.3 | 73.8 | Low SDI | Both |
| 2043 | 56.6 | 41.5 | 77.4 | Low SDI | Both |
| 2044 | 58.1 | 41.6 | 81.2 | Low SDI | Both |
| 2045 | 59.7 | 41.8 | 85.2 | Low SDI | Both |
| 2046 | 61.3 | 41.9 | 89.5 | Low SDI | Both |
| 2047 | 62.9 | 42.0 | 94.1 | Low SDI | Both |
| 2048 | 64.5 | 42.1 | 98.9 | Low SDI | Both |
| 2049 | 66.2 | 42.2 | 104.0 | Low SDI | Both |
| 2050 | 68.0 | 42.2 | 109.4 | Low SDI | Both |
| 2022 | 55.8 | 55.6 | 56.0 | Middle SDI | Male |
| 2023 | 56.6 | 56.0 | 57.3 | Middle SDI | Male |
| 2024 | 57.3 | 56.1 | 58.6 | Middle SDI | Male |
| 2025 | 58.0 | 56.1 | 60.0 | Middle SDI | Male |
| 2026 | 58.7 | 55.9 | 61.6 | Middle SDI | Male |
| 2027 | 59.3 | 55.6 | 63.3 | Middle SDI | Male |
| 2028 | 59.9 | 55.2 | 65.1 | Middle SDI | Male |
| 2029 | 60.6 | 54.7 | 67.0 | Middle SDI | Male |
| 2030 | 61.2 | 54.2 | 69.1 | Middle SDI | Male |
| 2031 | 61.8 | 53.6 | 71.3 | Middle SDI | Male |
| 2032 | 62.4 | 52.9 | 73.7 | Middle SDI | Male |
| 2033 | 63.0 | 52.2 | 76.1 | Middle SDI | Male |
| 2034 | 63.6 | 51.4 | 78.7 | Middle SDI | Male |
| 2035 | 64.2 | 50.6 | 81.5 | Middle SDI | Male |
| 2036 | 64.8 | 49.8 | 84.4 | Middle SDI | Male |
| 2037 | 65.4 | 48.9 | 87.5 | Middle SDI | Male |
| 2038 | 66.0 | 48.0 | 90.7 | Middle SDI | Male |
| 2039 | 66.6 | 47.1 | 94.0 | Middle SDI | Male |
| 2040 | 67.1 | 46.2 | 97.6 | Middle SDI | Male |
| 2041 | 67.7 | 45.3 | 101.3 | Middle SDI | Male |
| 2042 | 68.3 | 44.3 | 105.1 | Middle SDI | Male |
| 2043 | 68.8 | 43.4 | 109.2 | Middle SDI | Male |
| 2044 | 69.4 | 42.5 | 113.5 | Middle SDI | Male |
| 2045 | 70.0 | 41.5 | 117.9 | Middle SDI | Male |
| 2046 | 70.5 | 40.6 | 122.6 | Middle SDI | Male |
| 2047 | 71.1 | 39.6 | 127.4 | Middle SDI | Male |
| 2048 | 71.6 | 38.7 | 132.5 | Middle SDI | Male |
| 2049 | 72.1 | 37.7 | 137.8 | Middle SDI | Male |
| 2050 | 72.7 | 36.8 | 143.4 | Middle SDI | Male |
| 2022 | 129.3 | 128.9 | 129.6 | Middle SDI | Female |
| 2023 | 132.6 | 131.5 | 133.7 | Middle SDI | Female |
| 2024 | 135.9 | 133.6 | 138.2 | Middle SDI | Female |
| 2025 | 139.0 | 135.1 | 143.1 | Middle SDI | Female |
| 2026 | 142.2 | 136.1 | 148.5 | Middle SDI | Female |
| 2027 | 145.3 | 136.6 | 154.4 | Middle SDI | Female |
| 2028 | 148.4 | 136.7 | 161.0 | Middle SDI | Female |
| 2029 | 151.4 | 136.4 | 168.2 | Middle SDI | Female |
| 2030 | 154.5 | 135.6 | 176.1 | Middle SDI | Female |
| 2031 | 157.7 | 134.5 | 184.8 | Middle SDI | Female |
| 2032 | 160.8 | 133.1 | 194.3 | Middle SDI | Female |
| 2033 | 164.0 | 131.3 | 204.8 | Middle SDI | Female |
| 2034 | 167.2 | 129.3 | 216.2 | Middle SDI | Female |
| 2035 | 170.5 | 127.1 | 228.7 | Middle SDI | Female |
| 2036 | 173.8 | 124.6 | 242.4 | Middle SDI | Female |
| 2037 | 177.2 | 122.0 | 257.4 | Middle SDI | Female |
| 2038 | 180.6 | 119.1 | 273.7 | Middle SDI | Female |
| 2039 | 184.1 | 116.2 | 291.6 | Middle SDI | Female |
| 2040 | 187.6 | 113.1 | 311.2 | Middle SDI | Female |
| 2041 | 191.2 | 110.0 | 332.5 | Middle SDI | Female |
| 2042 | 194.9 | 106.7 | 355.9 | Middle SDI | Female |
| 2043 | 198.6 | 103.4 | 381.5 | Middle SDI | Female |
| 2044 | 202.4 | 100.1 | 409.6 | Middle SDI | Female |
| 2045 | 206.3 | 96.7 | 440.3 | Middle SDI | Female |
| 2046 | 210.3 | 93.3 | 473.9 | Middle SDI | Female |
| 2047 | 214.3 | 89.9 | 510.8 | Middle SDI | Female |
| 2048 | 218.4 | 86.5 | 551.3 | Middle SDI | Female |
| 2049 | 222.6 | 83.1 | 595.8 | Middle SDI | Female |
| 2050 | 226.8 | 79.8 | 644.6 | Middle SDI | Female |
| 2022 | 92.2 | 92.0 | 92.5 | Middle SDI | Both |
| 2023 | 94.2 | 93.4 | 95.0 | Middle SDI | Both |
| 2024 | 95.9 | 94.3 | 97.6 | Middle SDI | Both |
| 2025 | 97.6 | 94.7 | 100.5 | Middle SDI | Both |
| 2026 | 99.1 | 94.7 | 103.6 | Middle SDI | Both |
| 2027 | 100.5 | 94.4 | 107.0 | Middle SDI | Both |
| 2028 | 101.8 | 93.6 | 110.8 | Middle SDI | Both |
| 2029 | 103.1 | 92.6 | 114.9 | Middle SDI | Both |
| 2030 | 104.4 | 91.3 | 119.4 | Middle SDI | Both |
| 2031 | 105.6 | 89.7 | 124.3 | Middle SDI | Both |
| 2032 | 106.8 | 88.0 | 129.7 | Middle SDI | Both |
| 2033 | 108.0 | 86.0 | 135.6 | Middle SDI | Both |
| 2034 | 109.2 | 83.9 | 142.1 | Middle SDI | Both |
| 2035 | 110.3 | 81.6 | 149.1 | Middle SDI | Both |
| 2036 | 111.5 | 79.3 | 156.8 | Middle SDI | Both |
| 2037 | 112.7 | 76.9 | 165.1 | Middle SDI | Both |
| 2038 | 113.8 | 74.4 | 174.3 | Middle SDI | Both |
| 2039 | 115.0 | 71.8 | 184.2 | Middle SDI | Both |
| 2040 | 116.2 | 69.2 | 195.0 | Middle SDI | Both |
| 2041 | 117.4 | 66.6 | 206.7 | Middle SDI | Both |
| 2042 | 118.5 | 64.0 | 219.5 | Middle SDI | Both |
| 2043 | 119.7 | 61.4 | 233.5 | Middle SDI | Both |
| 2044 | 121.0 | 58.8 | 248.6 | Middle SDI | Both |
| 2045 | 122.2 | 56.3 | 265.2 | Middle SDI | Both |
| 2046 | 123.4 | 53.8 | 283.2 | Middle SDI | Both |
| 2047 | 124.6 | 51.3 | 302.9 | Middle SDI | Both |
| 2048 | 125.9 | 48.9 | 324.4 | Middle SDI | Both |
| 2049 | 127.2 | 46.5 | 347.8 | Middle SDI | Both |
| 2050 | 128.4 | 44.2 | 373.4 | Middle SDI | Both |

***Footnote***: SDI: Socio-demographic index.
